# An age-structured SEIR model for COVID-19 incidence in Dublin, Ireland with framework for evaluating health intervention cost

**DOI:** 10.1371/journal.pone.0260632

**Published:** 2021-12-07

**Authors:** Fatima-Zahra Jaouimaa, Daniel Dempsey, Suzanne Van Osch, Stephen Kinsella, Kevin Burke, Jason Wyse, James Sweeney

**Affiliations:** 1 Department of Mathematics & Statistics, University of Limerick, Limerick, Ireland; 2 School of Computer Science & Statistics, Trinity College Dublin, Dublin, Ireland; 3 Kemmy Business School, University of Limerick, Limerick, Ireland; Universitat Rovira i Virgili, SPAIN

## Abstract

Strategies adopted globally to mitigate the threat of COVID–19 have primarily involved lockdown measures with substantial economic and social costs with varying degrees of success. Morbidity patterns of COVID–19 variants have a strong association with age, while restrictive lockdown measures have association with negative mental health outcomes in some age groups. Reduced economic prospects may also afflict some age cohorts more than others. Motivated by this, we propose a model to describe COVID–19 community spread incorporating the role of age-specific social interactions. Through a flexible parameterisation of an age-structured deterministic Susceptible Exposed Infectious Removed (SEIR) model, we provide a means for characterising different forms of lockdown which may impact specific age groups differently. Social interactions are represented through age group to age group contact matrices, which can be trained using available data and are thus locally adapted. This framework is easy to interpret and suitable for describing counterfactual scenarios, which could assist policy makers with regard to minimising morbidity balanced with the costs of prospective suppression strategies. Our work originates from an Irish context and we use disease monitoring data from February 29th 2020 to January 31st 2021 gathered by Irish governmental agencies. We demonstrate how Irish lockdown scenarios can be constructed using the proposed model formulation and show results of retrospective fitting to incidence rates and forward planning with relevant “what if / instead of” lockdown counterfactuals. Uncertainty quantification for the predictive approaches is described. Our formulation is agnostic to a specific locale, in that lockdown strategies in other regions can be straightforwardly encoded using this model.

## 1 Introduction

The global race to manage the existential threat posed by COVID–19 has used non-pharmaceutical interventions (NPIs) such as lockdowns (or restriction of movement) measures as a central tenet. These will continue to play a major role in public health policy as new variants emerge and before a full vaccine roll-out has been reached. As nations have come to terms with COVID–19 throughout the past year and a half, the societal and economic impacts of the pandemic have become clear. A systemic shock to the world of work, widespread job losses in certain economic sectors, and a vast reduction in person-to-person social contact has given rise to an epoch of uncertainty, anxiety and fear. While older individuals are observed to be gravely threatened by the risk of infection, younger people have been particularly impacted by deteriorating mental health during this time [1] in addition to reduced economic prospects [2]. Governments have found themselves performing a difficult balancing act. Strict lockdown measures are necessary for public safety and to prevent health systems from becoming overwhelmed. However, periods of strict measures need to be punctuated by temporary easing of restrictions whenever possible to give hope to businesses and reduce the psycho-social demands placed on citizens. National “maps” and “road-plans” for emerging from COVID–19 that were proposed in the first quarter of 2020 by national Governments have been tweaked and revised world-wide; the time elapsed since March 2020 has been characterised by an ebb and flow of various forms of restriction of movement, both within and between nations.

It seems that lockdown measures and their consequences will be present in citizens’ lives for some time to come. Certain measures or guidelines to citizens may target specific age groups. For example, guidance has often urged extra protection for the elderly; in Ireland and the UK, this has been termed “cocooning”. There has been much debate about the risks posed by keeping schools for children open and as a result there has been variation in school closures globally. Quantifying the potential impact of new or changing measures that target age cohorts differently is thus essential. The exploration and consideration of counterfactuals can provide valuable lessons and insights to policy makers at a time of much uncertainty.

Our contribution in this article is to propose and calibrate a flexible model within the Susceptible Exposed Infected Recovered (SEIR) class, which can characterise different forms of lockdown measures with age structuring. The implied mortality burden of a lockdown architecture can be assessed through forward projection from the model. We show how this age-structured SEIR model, which includes explicit modelling of social mixing, can be used to assess and quantify the overall potential disease burden resulting from restriction of movement measures. Social mixing is modelled using age group to age group contact rates, allowing for assessment of the long run impact brought about by lockdowns which implicitly target specific age groups. The primary benefit of this approach is the potential to evaluate the ‘cost’ of specific intervention actions in terms of impacts on the general public. If each of the public health interventions that are being considered can be economically costed, then a strategy for disease suppression in tandem with minimising economic costs can be explored. As an example, forward projection can indicate costs to economic sectors such as the night–time economy which relies heavily on young adults. This view is not constrained to economic costs alone, as public health authorities may alternatively focus on health costs such as mental health impacts brought about by constraints on social mixing amongst the general public.

There has been some exploration of age-structured SEIR models for disease incidence. A SEIR model with age-structuring is used by [3] for the London area incorporating contact tracing; their interest was detailing the impact of social mixing and contact tracing on the effective reproduction rate of the disease as opposed to model calibration. In a similar vein, [4] use assumed epidemiological parameters to simulate the impact of age-specific control measures and contact tracing impact with a focus on the impact of control measures on factors including hospitalisations and deaths. The impact of four control measures (school closure, social distancing, quarantine, and isolation) are simulated by [5] to explore reproduction rates in South Korea using an age-structured SEIR model of disease spread. Maximum likelihood estimation is used to estimate contact scaling parameters, however no uncertainty in estimates or projections is presented. A two-cohort age-segmented model (age in years ≤65/>65) is proposed by [6] for Mexican incidence counts, suggesting that age specific control measures may have utility for public health policy decisions. The impact of three specific governmental interventions on case incidence is discussed by [7] employing an age-structured SEIR model with predetermined model parameters. A Susceptible Infected Recovered Dead model is fitted to data from Brazil by [8] examining how different interventions affect different age groups. They expand the model by including a hospitalisation compartment to project when demand on Intensive Care Units could exceed supply under different interventions. A SEIRD (SEIR with death compartment) model is used by [9] to project the number of deaths over the course of the vaccine rollout in the UK considering different lockdown scenarios. A Bayesian hierarchical model is used to estimate the effect of government interventions in [10], similar to previous work by [11] but harnessing data from multiple countries to disentangle the effects of different NPIs.

The dynamics of the age-structured SEIR model we propose have a number of advantages over these competing approaches. We account for the impact of movement restrictions on population mixing by scaling age-structured contact matrices, as with [12], however our scaling parameters are calibrated using the time series of observed Irish incidence counts as opposed to best-guess estimates. Where the parameters governing dynamics of models cannot be estimated due to data sparsity, we use recent results published in the COVID–19 literature on infection dynamics as well as expert opinion from the Irish Epidemiological Modelling Advisory Group (IEMAG); note that IEMAG developed an initial SEIR model [13] (see also [Gleeson et al., in press]) that we extend through the introduction of age-structuring and incorporation of the contact patterns, thus relaxing the assumption of homogeneous mixing across population age groups. This assumption implies that the force of infection is the same for all ages and may lead to the misrepresentation of disease dynamics for populations with heterogeneous population mixing and non-random contact patterns. The force of infection in our extended model reflects the age-related degree of mixing both within and among different age-groups and this is seen as a more realistic transmission hypothesis. We use a statistical bootstrapping approach to present uncertainties in learned parameters, in addition to providing uncertainty intervals for incidence projections.

The available data for calibration of social contacts consists of daily case counts and the specific lockdown measures implemented within Ireland from February 29th 2020 to January 31st 2021. While we present an analysis specific to an Irish context, we argue that the proposed approach is adaptable to other locales, wherein region specific macro-level behaviours can be calibrated. Furthermore, the framework we present can be easily adapted to incorporate more in depth population mixing knowledge from contact-tracing initiatives as well as allowing for estimation of all unknown model parameters.

Based on the SEIR model described in this article, we have developed an app that, given a user specified lockdown regime, creates an 8 week ahead forecast for estimated deaths and economic costs in Dublin. The app is written in the statistical programming language R version 4.1.1 [14] using the shiny package [15]. The interactive visuals produced by the app are created using the plotly package [16] in conjunction with the ggplot2 package [17]. The methodology and software used to produce the output is described in section 4, and is freely available at https://github.com/fatimZJ/Covid-19-Project.

The remainder of the paper is organized as follows. Section 2 provides an overview of observed COVID–19 incidence data and lockdown architectures in Ireland, a model of the effects of these lockdowns on social mixing by rescaling the population contact matrix over time, and the associated economic impacts. In Section 3 we present our proposed age-structured SEIR model which incorporates the scaled elements of the contact matrix, and, hence, lockdown effects. Section 4 describes calibration of the free parameters in the model, and quantification of uncertainty using a bootstrapping approach. Section 5 outlines the results of retrospective model fitting to Irish data, with associated projections and examples of some interesting counterfactual situations provided in Section 6. We conclude in Section 7 with a discussion.

## 2 Data sources

We restrict our data sources to those that are typically freely publicly available, allowing for ease of implementation in other regions. The available data in an Irish context consists of daily incidence counts and the dates of changes of lockdown restrictions. Estimated contact matrices for age structured population mixing are sourced from literature. We defer discussion of the mechanistic parameters sourced from the COVID–19 literature to Section 3.

### 2.1 Daily incidence counts

**Fig 1:**
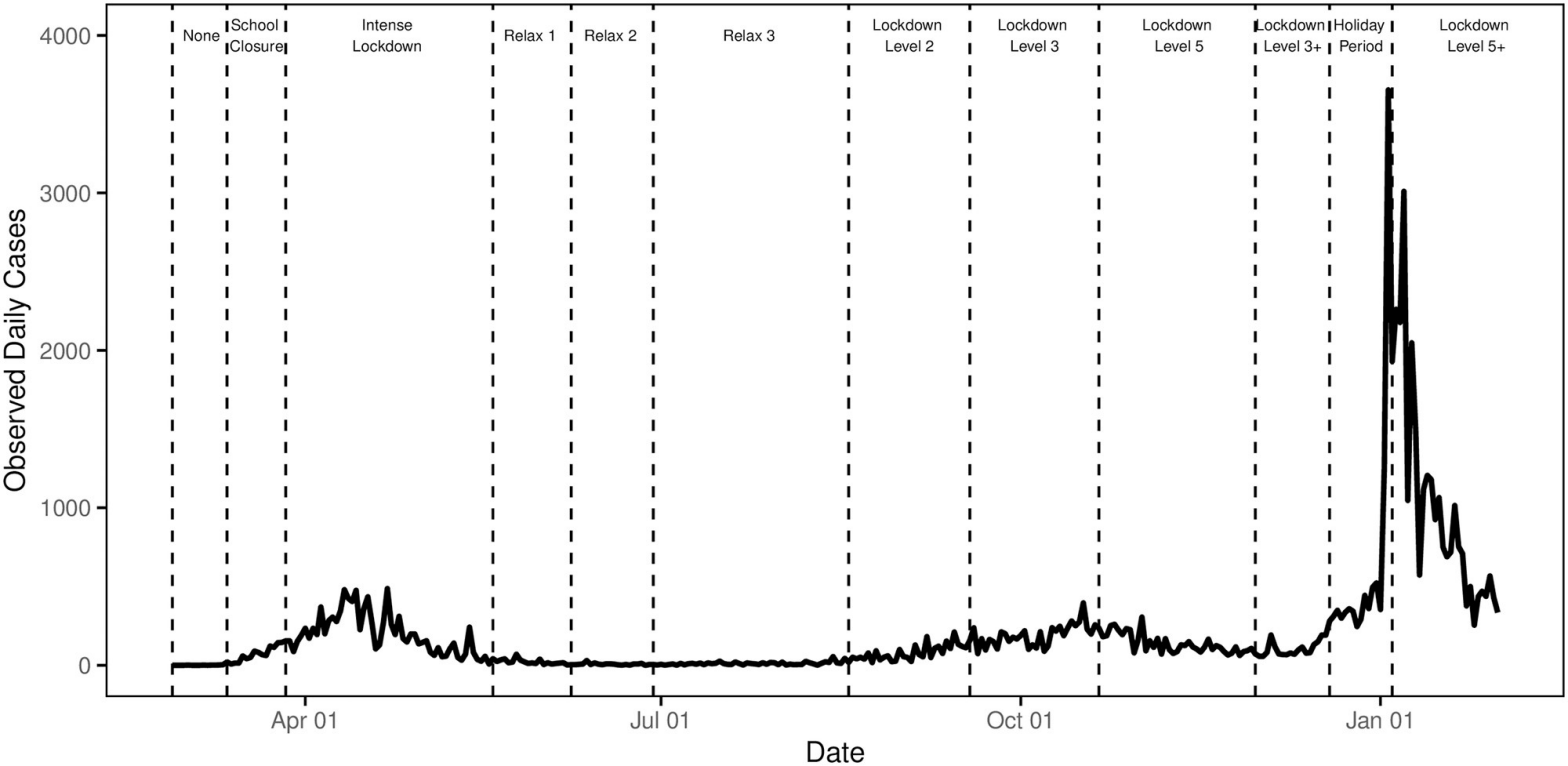
COVID–19 daily case incidence with corresponding lockdown levels in each period between February 2020 and January 2021. Descriptions of individual lockdown levels are presented in S1 Table in S1 File.

The Irish Health Surveillance Protection Centre (data.gov.ie) provide anonymised daily COVID–19 incidences. We use the data from the period of February 29th 2020 to January 31st 2021 for model calibration. The daily COVID–19 count incidence is shown in Fig 1. Age structured case count data is not publicly available in Ireland, and hence we use aggregate case counts at the population level. We use projected population data for 2019 provided by Irish Central Statistics Office (CSO) (https://data.cso.ie/table/PEB07) to estimate the age-structured population breakdown by county to estimate Dublin’s population. Given the constraints on public movement, we make the assumption that the 2019 projections are representative of the population since the beginning of the pandemic.

### 2.2 Form of lockdown restrictions

Fig 1 shows the timeline and duration of varying degrees of restriction measures (vertical dashed lines) implemented in Ireland from March 2020. In line with [13], we define the 28th February 2020 as “day zero” of the Irish epidemic. As with many other countries, the Irish government introduced a strict lockdown in the early stages of the pandemic which lasted until May 2020. This lockdown was followed by a gradual easing of restrictions throughout the summer until case numbers began to rise in early autumn, when harsher restrictions were reintroduced and another phase of a strict lockdown was announced for late October. Restrictions were eased over the month of December but a subsequent wave of cases forced the implementation of a further strict lockdown immediately after the December holiday period. A more detailed overview of the restrictions is provided in S1 Table in Appendix A of S1 File.

The nature of restrictions on public mobility in Ireland, announced by the Irish government in April 2020, follow five levels. Level one is the least restrictive with this increasing to most restrictive at level five. In level one, food venues and bars remain open, gatherings of up to fifty people are permitted outdoors and sporting events can take place with restrictions on numbers. Level five corresponds to a near total blanket close on all activities. As the public health situation evolved during 2020, small adjustments were made to these levels with slight easing of targeted restrictions (for example, reopening of schools or childcare) within more severe lockdowns. An overview of the five level lockdowns is provided in Table 1 with in-depth detail available at gov.ie/en/campaigns/resilience-recovery-2020–2021-plan-for-living-with-covid-19/. We denote the time intervals of lockdown measures using $I_{k}=\left(r_{k},r_{k+1}\right]$, where *r*_*k*_ is the time of the beginning of the *k*th regime for *k* = 1, …, *N* where *N* = 12, with the first regime corresponding to no intervention from 29th February to 11th March 2020. Thus, we define *r*_1_ = 0 corresponding to 29th February 2020 and *r*_*N*+1_ = 336 corresponding to 31th January 2021.

**Table 1:** Summary overview of the restrictions impacting on public gatherings for each of the five lockdown levels in Ireland. The numbers comprise the limits on individuals allowed to gather together in each social setting unless otherwise specified as a household limit. Details on other restrictions, such as on private travel, have been omitted for brevity.

	Level 1	Level 2	Level 3	Level 4	Level 5
**House visits**	10 (3 households)	6 (2–3 households)	1 household	0	0
**Gatherings**	15 outdoor	6 indoor 50 outdoor	0	0	0
**Weddings**	100	50	25	6	6
**Indoor events**	100	50	0	0	0
**Sporting events**	100 indoor 200 outdoor	50 indoor 100 outdoor	0	0	0
**Food venues**	Open	6 (3 households)	15 outdoor	15 outdoor	0
**Pubs**	Open	6 (3 households)	15 outdoor	15 outdoor	0
**Public transport capacity**	100%	50%	50%	25%	25%

### 2.3 Age structuring and social mixing

COVID–19 is an airborne virus, hence consideration of close social mixing in the population is essential to capturing the observed patterns of infection. Furthermore, the strong association of morbidity patterns with the elderly, and more recently younger persons [18], suggest consideration of age structured social mixing will be a key component of future projections [6, 12]. Age-structured social mixing is typically captured in SEIR models through the use of age group to age group contact matrices. Although such matrices cannot capture the granular complexity of individual human interactions, they provide a reasonable approximation that can be incorporated into mathematical models for infectious diseases as demonstrated by [19], and within this article. We follow [12] and stratify the population into five-year bands from age 0 up to age 75, with one category for all individuals aged 75 and above, giving *A* = 16 age groups.

**Fig 2:**
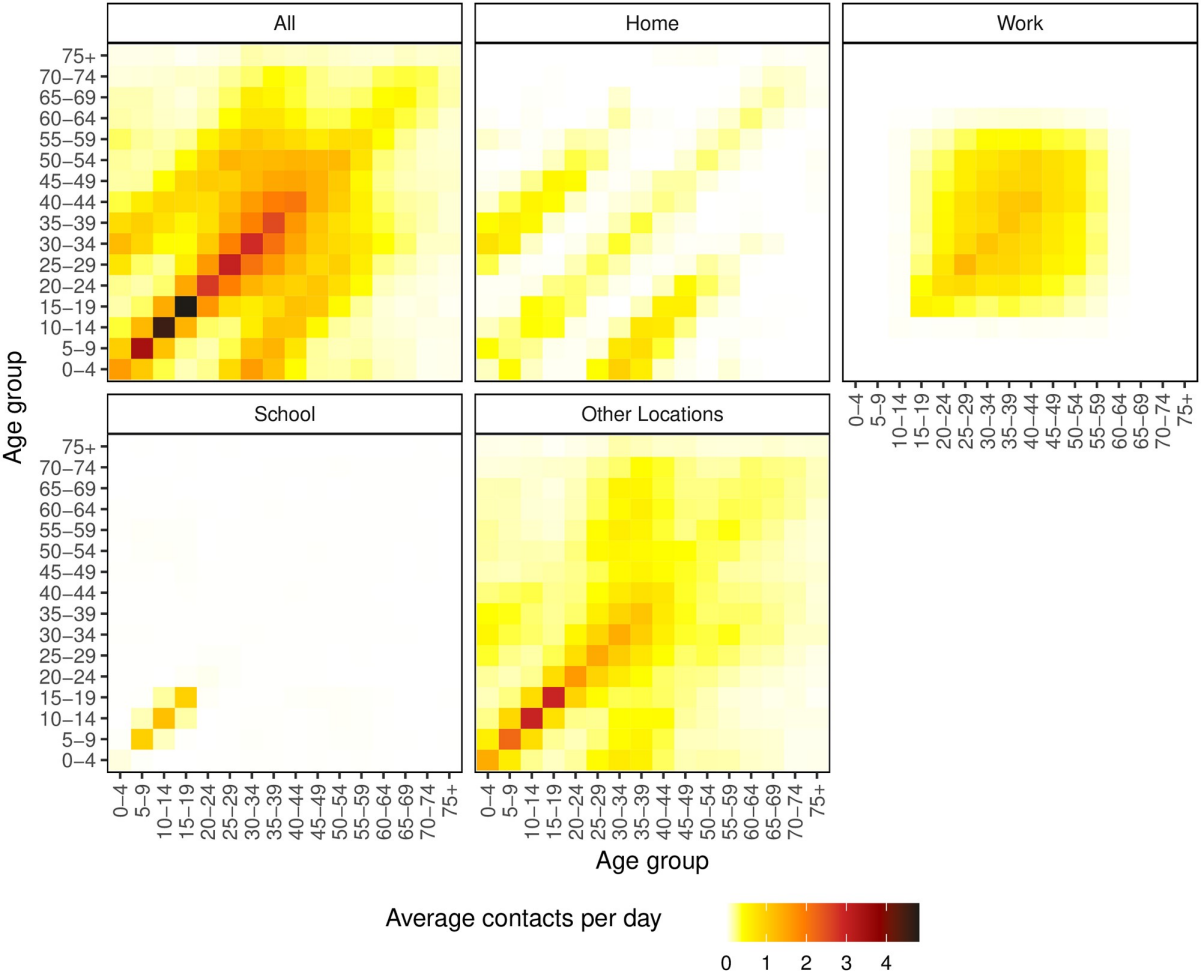
Estimated social contact matrices for Irish population mixing at 5 year intervals [21].

Contact tracing has been a prominent factor in disease suppression in Asian countries to date. However, such data is not available in an Irish context, and we are unaware of any large-scale survey or study on age-structured social mixing patterns in Ireland. However, [20, 21] provide a methodology for deriving contact patterns by leveraging mixing patterns studied in other European countries. Our analysis relies on contact matrices given by [21] who projected age and location specific contact matrices in 16 age bands for 152 countries including Ireland. These are constructed from the POLYMOD study [19], which incorporated large-scale demographic household surveys (from the UN population division) and school and labour force participation rates. The estimated Irish contact interactions are shown in Fig 2. For the purpose of our work, we sum together expected contacts in the home, work, school and other locations to give an overall matrix of expected contacts (‘All’ in Fig 2). We assume that the Irish contact matrix applies to just Dublin.

Government interventions to suppress virus spread result in changes in population mixing. Therefore, to reflect these changes, we introduce a free parameter, *θ*_*k*_, which scales the aggregate expected contact matrix for the time interval $I_{k}=\left(r_{k},r_{k+1}\right]$ corresponding to the *k*th of *N* lockdown regimes. The aforementioned age-structured contact matrices are formed by entries *c*_*ij*_ representing the average number of daily contacts between an individual in age category *i* with an individual in age category *j*, where *i*, *j* = 1, …, *A*. Then, at time $t\in I_{k}$ (i.e., during the *k*th lockdown), the scaled contact matrix is

$$\begin{array}{c} (\begin{array}{ccc} \theta_{k}c_{1\;1} & \ldots & \theta_{k}c_{1\;A}\\ \vdots & \ldots & \vdots \\ \theta_{k}c_{A\;1} & \ldots & \theta_{k}c_{A\;A} \end{array})=\theta_{k}C,\;k=1,\ldots ,N. \end{array}$$

In Section 3 we outline the estimation of the scaling parameters ***θ*** = (*θ*_1_, …, *θ_N_*) for each lockdown period using observed incidences in Dublin. This allows an estimation of macro-level behavioural changes in socialising brought about by specific measures. We explore the robustness of the model results presented in Section 5 to the matrix **C** by exploring three alternative specifications of this matrix in SC Appendix.

### 2.4 Economic cost of lockdown measures

Our age-structured modelling approach offers the potential for evaluation of age-related economic or health costs in tandem with public morbidity. Here, we explore costing economic impacts of different lockdown measures used by the Irish government to date. There has been much interest in the economic impact of COVID–19, with a rapidly expanding literature. For example, [22] study the basic macroeconomics of epidemics while [23] attempt a partial literature review of the main approaches to macroeconomic costing of the epidemic to date. [24] extend the canonical macroeconomic framework to SEIR models of the type we develop in this paper. [25] produce initial estimates of the medium-term impact of the crisis on the Irish economy. Relating to Ireland, we draw on the work of [26] in order to construct weekly estimates of year on year (same week in different years) growth rate of Gross Domestic Product (GDP). This existing approach makes forecasts based on a neural network modelling framework, with training of the model done via quarterly GDP and Google Trends search intensity data gathered over forty six countries from the beginning of 2005 for sixty one quarters. The quarterly data is used to construct a forecasting model corresponding to a weekly resolution making an assumption of frequency neutrality [26].

While there are obvious criticisms of using GDP as a sound economic measure, here we employ it as a proxy to give a high level view of implied costs of lockdown to the economy. In this frame of reference, we can justify the approximation in three ways. Firstly, GDP is an aggregate flow measure containing values in euros of expenditures by households, firms, and government, particularly the importing, and exporting parts of the Irish economy [27]. The weekly GDP tracker combines the sometimes countervailing microeconomic elements of the COVID crisis into a single variable. While expenditures by the household and corporate sectors was reduced due to government restrictions, the government expanded its support activities in terms of direct payments to furloughed workers, turnover replacements and generous liquidity packages for firms, debt and tax arrears-warehousing, and increases in direct spending on health-related measures [28]. Secondly, it is acknowledged that Ireland is a small open economy with a very large multinational sector relative to other countries of similar size and development level. While GDP is understood for example by [29] as being a relatively poor measure of the health of the Irish economy due to the influence of large multinational companies on the economy, these companies, particularly in the pharmaceutical and ICT sectors, helped keep the government’s budget deficit lower than it otherwise would have been. Finally, the weekly GDP calculations of [26] enable the production of a measure of the overall economic impact of COVID–19 relative to each lockdown period. A weekly tracker of economic activity allows for matching of economic predictions with the timescale of typical lockdown duration. The generation of in-model behavioural elasticities using a panel of 46 countries across several decades also means the model has the ability to react within a period of crisis, as the economy’s participants learn from the first lockdown experience, and adapt themselves to cope with further lock downs.

## 3 SEIR model specification

Irish population modelling of COVID–19 during the crisis has been carried out by the Irish Epidemiological Modelling and Advisory Service (IEMAG) [13]. They present a model for the Irish population where, at any point in time, an individual is assumed to be in one of a number of distinct model compartments that describe COVID–19 status. Movement between compartments over time is based on the current understanding of the epidemiology of COVID–19, as evidenced by the extensive literature review and evidence synthesis conducted by [30–32].

We evolve this model to consider age-structured differences in population mixing. We assume closed age classes, such that population *N*_*i*_ of age class *i* is the sum of susceptible (*S*_*i*_), exposed (*E*_*i*_), infected and removed (*R*_*i*_) compartments for that age class. There is no movement between age classes. Infected cases fall into a number of compartments: asymptomatic ($I_{i}^{\text{AS}}$), pre-symptomatic ($I_{i}^{\text{PS}}$), symptomatic and self-isolating without testing ($I_{i}^{\text{SI}}$), symptomatic and awaiting test results ($I_{i}^{\text{ST}}$), symptomatic and isolating after receiving positive test results ($I_{i}^{\text{PI}}$) and symptomatic but not tested or isolating ($I_{i}^{\text{SN}}$). The closed age class assumption implies that

$$\begin{array}{c} N_{i}=S_{i}+E_{i}+I_{i}^{\text{AS}}+I_{i}^{\text{PS}}+I_{i}^{\text{SI}}+I_{i}^{\text{ST}}+I_{i}^{\text{PI}}+I_{i}^{\text{SN}}+R_{i},\;i=1,\ldots ,A. \end{array}$$

Exposed individuals are those incubating the disease but not yet infectious. Asymptomatic individuals are infectious but do not exhibit symptoms. Pre-symptomatic individuals are infectious but have yet to show symptoms. As pre-symptomatic individuals’ symptoms develop, they will move to one of the infectious or symptomatic compartments, either self isolating and following government guidance around testing, or neither getting tested nor isolating when symptomatic, i.e., ignoring symptoms. Following infection, individuals move to the removed class (*R*_*i*_), which accounts for cases who recover and those who die.

**Fig 3:**
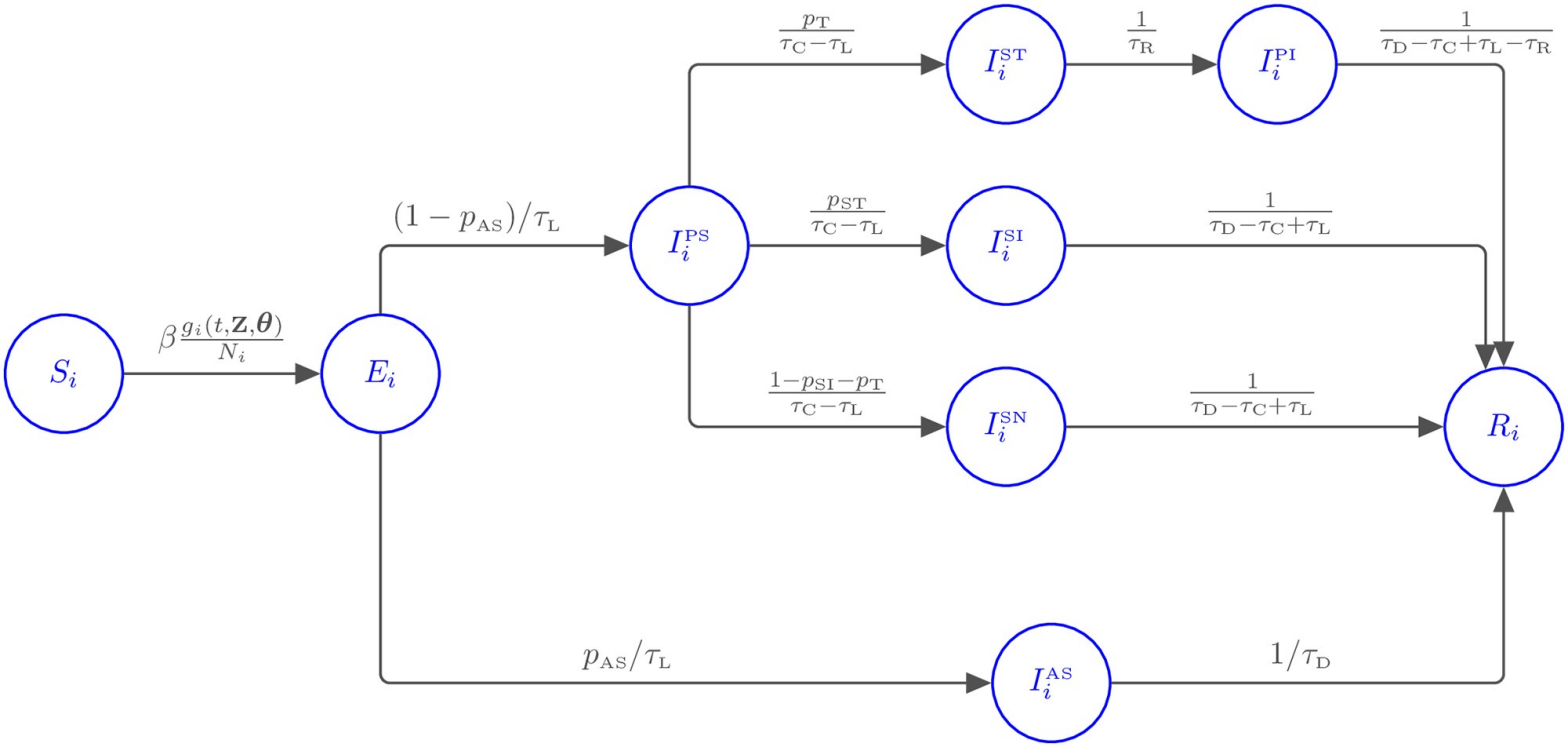
Diagram representing interactions in the age-structured SEIR system of ODEs with rate of movement between classes indicated. The compartments are susceptible (*S*), exposed (*E*), infected and removed (*R*), asymptomatic (*I*^AS^), pre-symptomatic (*I*^PS^), symptomatic and self-isolating without testing (*I*^SI^), symptomatic and awaiting test results (*I*^ST^), symptomatic and isolating after receiving positive test results (*I*^PI^) and symptomatic but not tested or isolating (*I*^SN^). A full description is given in Eq (1).

We write the system of ordinary differential equations (ODEs) describing the SEIR model for age class *i* = 1, …, *A*:

$$\begin{array}{c} \begin{array}{cc} & \begin{array}{cc} & \frac{dS_{i}}{d\;t}=-\beta \;\frac{g_{i}\left(t,z,\theta \right)}{N_{i}}\;S_{i}\\ & \frac{dI_{i}^{\text{AS}}}{d\;t}=p_{\text{AS}}\;\frac{E_{i}}{\tau_{\text{L}}}\;-\;\frac{I_{i}^{\text{AS}}}{\tau_{\text{D}}}\\ & \frac{dI_{i}^{\text{SI}}}{d\;t}=p_{\text{SI}}\;\frac{I_{i}^{\text{PS}}}{\tau_{\text{C}}-\tau_{\text{L}}}\;-\;\frac{I_{i}^{\text{SI}}}{\tau_{\text{D}}-\tau_{\text{C}}+\tau_{\text{L}}}\\ & \frac{dI_{i}^{\text{SN}}}{d\;t}=\left(1-p_{\text{SI}}-p_{\text{T}}\right)\;\frac{I_{i}^{\text{PS}}}{\tau_{\text{C}}-\tau_{\text{L}}}\;-\;\frac{I_{i}^{\text{SN}}}{\tau_{\text{D}}-\tau_{\text{C}}+\tau_{\text{L}}} \end{array}\;\;\begin{array}{cc} & \frac{dE_{i}}{d\;t}=\beta \;\frac{g_{i}\left(t,z,\theta \right)}{N_{i}}\;S_{i}-\frac{E_{i}}{\tau_{\text{L}}}\\ & \frac{dI_{i}^{\text{PS}}}{d\;t}=\left(1-p_{\text{AS}}\right)\;\frac{E_{i}}{\tau_{\text{L}}}\;-\;\frac{I_{i}^{\text{PS}}}{\tau_{\text{C}}-\tau_{\text{L}}}\\ & \frac{dI_{i}^{\text{ST}}}{d\;t}=p_{\text{T}}\;\frac{I_{i}^{\text{PS}}}{\tau_{\text{C}}-\tau_{\text{L}}}\;-\;\frac{I_{i}^{\text{ST}}}{\tau_{\text{R}}}\\ & \frac{dI_{i}^{\text{PI}}}{d\;t}=\frac{I_{i}^{\text{ST}}}{\tau_{\text{R}}}\;-\;\frac{I_{i}^{\text{PI}}}{\tau_{\text{D}}-\tau_{\text{C}}+\tau_{\text{L}}-\tau_{\text{R}}} \end{array}\\ & \;\;\frac{dR_{i}}{d\;t}=\frac{I_{i}^{\text{AS}}}{\tau_{\text{D}}}\;+\;\frac{I_{i}^{\text{SI}}}{\tau_{\text{D}}-\tau_{\text{C}}+\tau_{\text{L}}}\;+\;\frac{I_{i}^{\text{PI}}}{\tau_{\text{D}}-\tau_{\text{C}}+\tau_{\text{L}}-\tau_{\text{R}}}\;+\;\frac{I_{i}^{\text{SN}}}{\tau_{\text{D}}-\tau_{\text{C}}+\tau_{\text{L}}} \end{array} \end{array}$$ (1)

where the function *g_i_*(*t*, **z**, ***θ***) in the mass relation for being exposed when susceptible is

$$\begin{array}{c} g_{i}\left(t,z,\theta \right)=\sum_{k=1}^{N}\;I(\;t\in I_{k}\;)\;\sum_{j=1}^{A}\;\theta_{k}\;c_{ij}\;[\;\alpha \;I_{j}^{\text{AS}}+I_{j}^{\text{PS}}+\kappa \;I_{j}^{\text{SI}}+I_{j}^{\text{ST}}+\kappa \;I_{j}^{\text{PI}}+I_{j}^{\text{SN}}\;] \end{array}$$

where **z** denotes the entire state vector

$$\begin{array}{c} z=(\;S_{1},E_{1},\ldots ,S_{2},E_{2},\ldots ,S_{A},E_{A},\ldots ,R_{A}\;), \end{array}$$

and $I(\;t\in I_{k}\;)$ is an indicator function which equals one when $t\in I_{k}$ and is zero otherwise. A graphical illustration of the model is presented in Fig 3. Susceptible individuals in age class *i* are exposed to the virus through contacts with infected individuals in all age classes and this is described through the function *g_i_*(*t*, **z**, ***θ***). The level of exposure is modulated by the scaled average number of daily contacts with each age class, with a scaling factor for each lockdown regime. The parameters *α* and *κ* are used to describe transmission dynamics with respect to the various infected compartments. The reduction of transmission due to being asymptomatic is given by *α* which is taken as 0.55 [33]. Individuals in isolation are expected to have a reduced infectious burden on those they interact with. This is described using *κ* = 0.05 [13] and we assume an identical reduced burden from those who are positive and isolating.

The SEIR model makes extensive use of *β*, the multiplicative force of infection. The value of *β* is chosen based on a specified value of *R*_0_, the baseline reproduction rate. Baseline here highlights that this is the expected reproduction rate in Dublin, with a fully susceptible population (i.e., *S*_*i*_ = *N*_*i*_, *i* = 1, …, *A*), under no intervention. *R*_0_ can be expressed as the largest absolute eigenvalue of the next generation matrix, **Q** = **F** **V**^−1^ [34], where **F** and **V** are block matrices which describe the transmissions and the transitions between compartments, respectively. The analytic form of these matrices is given in Appendix B in S1 File. Factoring *β* out of the **F** matrix, $F=\beta \;\hat{F}$, the dominant eigenvalue of **Q** can be expressed as a product of *β* and the dominant eigenvalue of $\hat{F}\;V^{-1}$. Hence, if *R*_0_ is determined and *β* is desired, the expression can easily be rearranged to give *β* as follows:

$$\begin{array}{c} \beta =R_{0}/\xi \end{array}$$ (2)

where *ξ* is the largest eigenvalue of $\hat{F}\;V^{-1}$. Note, the values of *R*_0_ and *β* are only calculated once as they are baseline values; shifts in infection dynamics away from the baseline are captured by ***θ***. Our choice for *R*_0_ is 3.4, based on [35]. This leads to *β* = 0.031 when not under intervention. A full list of parameter value settings used in our modelling is given in S3 Table in S1 File. We note here that the *θ*_*k*_, *k* = 1, …, *N* are unknown. The next section describes how these are estimated using observed case incidences.

## 4 Model fitting

Specification of the model in Section 3 uses parameters ***θ*** = (*θ*_1_, …, *θ_N_*) to rescale, for each of the *N* = 12 lockdown intervention policies, what would have been the assumed average contacts between individuals in the various age classes under normal circumstances (i.e., prior to the pandemic). Estimation of these parameters is of interest in predicting behaviour during lockdown, and hence for forecasting the benefit of specific interventions. Note that the remaining parameters (i.e., those other than ***θ***) on which the dynamics of the SEIR model (1) depend, describing the flow of individuals between compartments, are based on expert opinion [13, 35]. The data currently available is not sufficiently rich to estimate these. We first describe estimation of ***θ*** and then a parametric bootstrap method [36] to provide uncertainty, which can be propagated through model forecasts.

In order to link the model with observed data we monitor the cumulative number of cases up to time *t* for each age class. It is only cases exiting the $I_{i}^{\text{ST}},i=1,\ldots ,A$ compartment that can be linked to observed incidence counts in the general population. We can think of a variable, counting infected cases as they exit compartment $I_{i}^{\text{ST}}$ before going into $I_{i}^{\text{PI}}$. For scaling ***θ***, age class *i* and time *t*, denote this by *X_i_*(*t*; ***θ***). This can be related to the other model compartments through

$$\begin{array}{c} \frac{dX_{i}}{d\;t}=\frac{I_{i}^{\text{ST}}}{\tau_{\text{R}}},\;i=1,\ldots ,A. \end{array}$$ (3)

To compare outputs from the SEIR model with observed data, we use this count aggregated over age classes:

$$\begin{array}{c} X\left(t;\theta \right)=\sum_{i=1}^{A}X_{i}\left(t;\theta \right) \end{array}$$ (4)

which gives total cumulative case counts to time *t*. Evaluating this at *t*_*d*_ = *hd*, *d* = 1, …, *n* where *h* generates a time discretisation corresponding to consecutive days, we can then compare *X*(*t_d_*; ***θ***) to observed cumulative cases at day *d*. We denote the observed cumulative counts at day *d* by *x*_*d*_.

### 4.1 Estimation of regime specific contact scaling parameters

To estimate ***θ***, we minimize the squared error loss, i.e., the residual sum of squares,

$$\begin{array}{c} RSS\left(\theta \right)=\sum_{d=1}^{n}{\left(x_{d}-X\left(t_{d};\theta \right)\right)}^{2} \end{array}$$

on cumulative case counts. Minimization is carried out using the default Nelder-Mead alogrithm [37] provided in the R package optimx [38]. Note that each step of this algorithm, corresponding to a proposed ***θ*** vector, requires the calculation of *X*(*t_d_*; ***θ***) to evaluate the suitability of ***θ*** through *RSS*(***θ***). In order to obtain *X*(*t_d_*; ***θ***), the system of ODEs given in (1) are numerically solved using the R package deSolve [39]. Specifically, we have found the lsoda function within this package to be particularly flexible, providing automatic selection of stiff or non-stiff methods; see [38] for details. When solving the system of ODEs for a candidate ***θ***, we take $I_{i}^{\text{PS}}=1/A$, *E*_*i*_ = 15/*A* and hence *S*_*i*_ = *N*_*i*_ − 16/*A* as the initial values for *i* = 1, …, *A*, as per [13]. The initial values for the remaining compartments are set to 0. Multiple random initialisations of ***θ*** are used to improve robustness of the overall algorithm with respect to the issue of convergence to local minima. When generating these initial vectors, we assume that the effect of lockdown measures is to reduce social mixing below pre-pandemic levels, and, therefore, use a *U*(0, 1) draw to initialise each parameter, i.e., $\theta_{k}^{0}∼U\left(0,1\right),k=1,\ldots ,N$. A summary of our estimation procedure is given in Algorithm 1.

**Algorithm 1** Estimation of ***θ***

1: **procedure** estimation *M*, *x*_*d*_, *d* = 1, …, *n*

2:  **for** *m* = 1, …, *M* **do**

3:   $\theta_{k}^{0(m)}∼\text{Unif}\left(0,1\right)$, *k* = 1, …, *N*

4:   $RSS\left(\theta \right)≔\sum_{d=1}^{n}\left(x_{d}-X\left(t_{d};\theta \right)\right)$ with *X*(*t_d_*; ***θ***) given by lsoda

5:   ${\hat{\theta }}^{\left(m\right)}={\text{argmin}}_{\theta }RSS\left(\theta \right)$ using optimx initialised at ***θ***^0(*m*)^

6:   $RSS^{\left(m\right)}=RSS\left({\hat{\theta }}^{\left(m\right)}\right)$

  **return** $\hat{\theta }=\left\{{\hat{\theta }}^{\left(m\right)}|RSS^{\left(m\right)}=\text{min}\left(RSS^{\left(1\right)},\ldots ,RSS^{\left(M\right)}\right)\right\}$

Since we have to solve the ODEs (1) numerically using lsoda of each iteration within the optimx optimisation, the above procedure is computationally intensive. On average it takes approximately 28 minutes to run the optimisation for each random initialisation on an Intel Core i5–8250U CPU with 4 cores. We use *C* = 300 random initialisations. Thus, to improve the computational feasibility, we have run initialisations in parallel on an EC2 instance hosted by Amazon Web Services with 32 cores and 64 GB memory.

The publicly available Irish data consists of case counts aggregated across all age classes at present. If more granular age data is available, a modification to the residual sum of squares objective above can be made. For example, if all *A* age classes as prescribed here are publicly available such that we can access *x*_*di*_, the observed cumulative counts at day *d* for age class *i* = 1, …, *A*, the objective

$$\begin{array}{c} RSS\left(\theta \right)=\sum_{d=1}^{n}\sum_{i=1}^{A}{\left(x_{di}-X_{i}\left(t_{d};\theta \right)\right)}^{2} \end{array}$$

could instead be used.

### 4.2 Propagating uncertainty in contact scaling parameters

We explore uncertainty in the estimation of ***θ*** and investigate how this propagates into the reproductive rate. In order to quantify uncertainty we follow [40] by using a parametric bootstrap approach. This approach makes use of an assumed generative parametric model for daily case counts based on the observed case counts. This model is then used to re-generate *B* synthetic instances of the daily new case series; each of these instances is used to re-estimate the vector ***θ***. The resulting empirical distribution of the re-estimated vectors can be used as an approximation to the sampling distribution of $\hat{\theta }$ (the estimate based on the original cumulative case counts).

The estimate $\hat{\theta }$ is found using the observed daily cumulative counts as described in Section 4.1. Given this estimate, the expected daily case count ${\hat{\mu }}_{d}$ for day *d* can be predicted using

$$\begin{array}{c} {\hat{\mu }}_{d}=X\left(t_{d};\hat{\theta }\right)-X\left(t_{d-1};\hat{\theta }\right),\;d\geq 1 \end{array}$$

where *t*_0_ ≔ 0 and *X*(0; ***θ***) ≔ 0. We assume a negative binomial distribution [40] as a generative model for daily case counts $Y_{d}∼\text{NegBin}\left({\hat{\mu }}_{d},\rho \right)$ with expected value ${\hat{\mu }}_{d}$ and dispersion parameter *ρ*:

$$\begin{array}{ccc} \text{Pr}(Y_{d}=y) & = & \frac{\Gamma (\rho +y)}{y!\;\Gamma (\rho )}{\left(\frac{{\hat{\mu }}_{d}}{{\hat{\mu }}_{d}+\rho }\right)}^{y}{\left(1+\frac{{\hat{\mu }}_{d}}{\rho }\right)}^{-\rho } \end{array}$$ (5)

where we have parameterised the negative binomial distribution through its expected value and dispersion. The value of *ρ* used for generating bootstrap *Y*_*d*_ series is the maximum likelihood estimate $\hat{\rho }$ based on the observed daily cases.

For each of *b* = 1, …, *B* bootstrap replications, we generate daily counts $y_{d}^{\left(b\right)}$ and convert to cumulative counts $x_{d}^{\left(b\right)},\;d=1,\ldots ,n$. Then the method of Section 4.1 is applied to the $x_{d}^{\left(b\right)}$ to produce a bootstrap estimate ${\hat{\theta }}^{\left(b\right)}$. Collectively, the *B* estimates ${\hat{\theta }}^{\left(b\right)}$ provide an approximation to the sampling distribution of $\hat{\theta }$. The steps are summarized in Algorithm 2.

**Algorithm 2** Parametric bootstrapping

**procedure** bootstrap(*B*, $\hat{\rho }$, ${\hat{\mu }}_{d},d=1,\ldots ,n$)

 **for** *b* = 1, …, *B* **do**

  $x_{0}^{\left(b\right)}≔0$

  **for** *d* = 1, …, *n* **do**

   $Y_{d}∼\text{NegBin}\left({\hat{\mu }}_{d},\hat{\rho }\right)$

   $x_{d}^{\left(b\right)}=x_{d-1}^{\left(b\right)}+y_{d}$

 ${\hat{\theta }}^{\left(b\right)}$ obtained from Algorithm 1

 **return** Bootstrap sample $\left\{{\hat{\theta }}^{\left(b\right)},1,\ldots ,B\right\}$

## 5 Results

In this section we present the results of fitting the age-structured SEIR model to Irish data using the methodology described in Section 4, and also discuss some economic findings.

### 5.1 Fitted SEIR model

**Fig 4:**
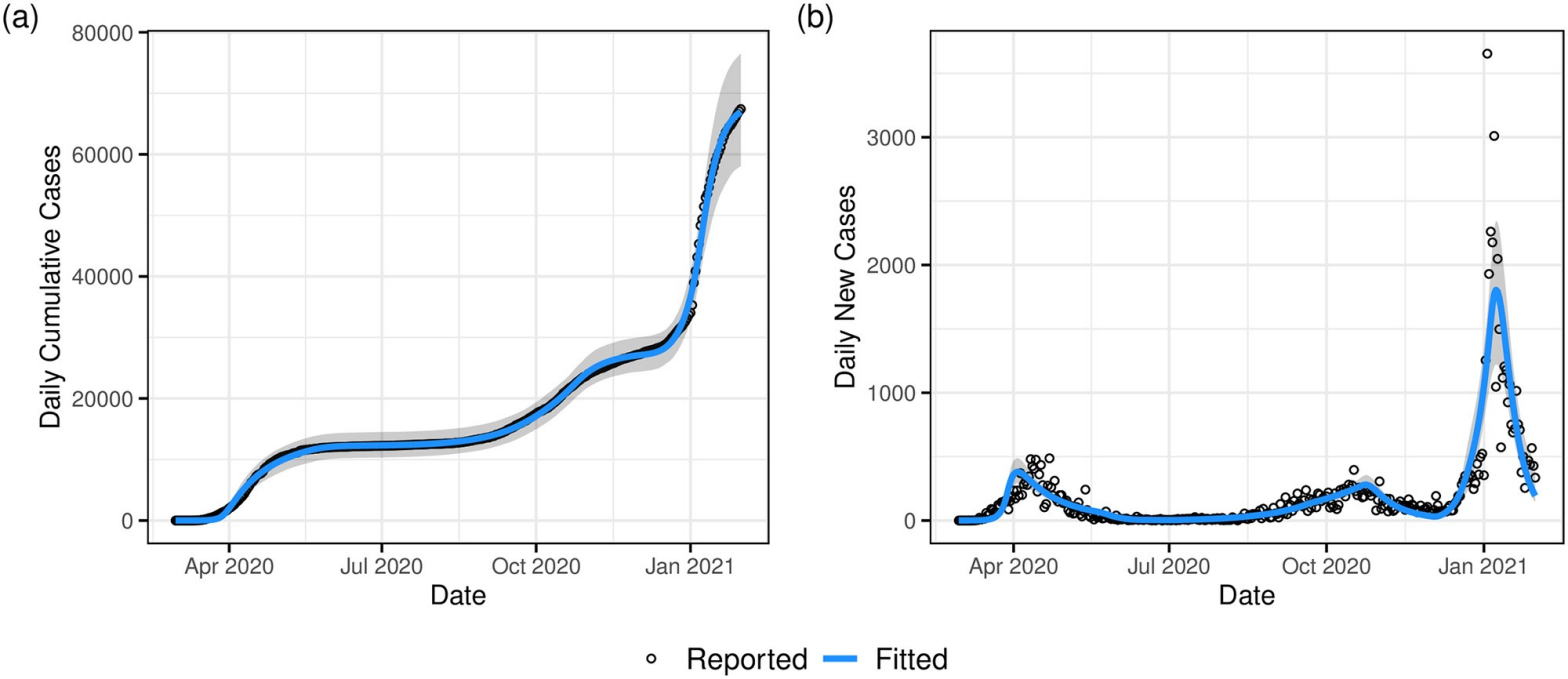
Model fit to daily recorded cases with bootstrapped 95% uncertainty bounds.

**Fig 5:**
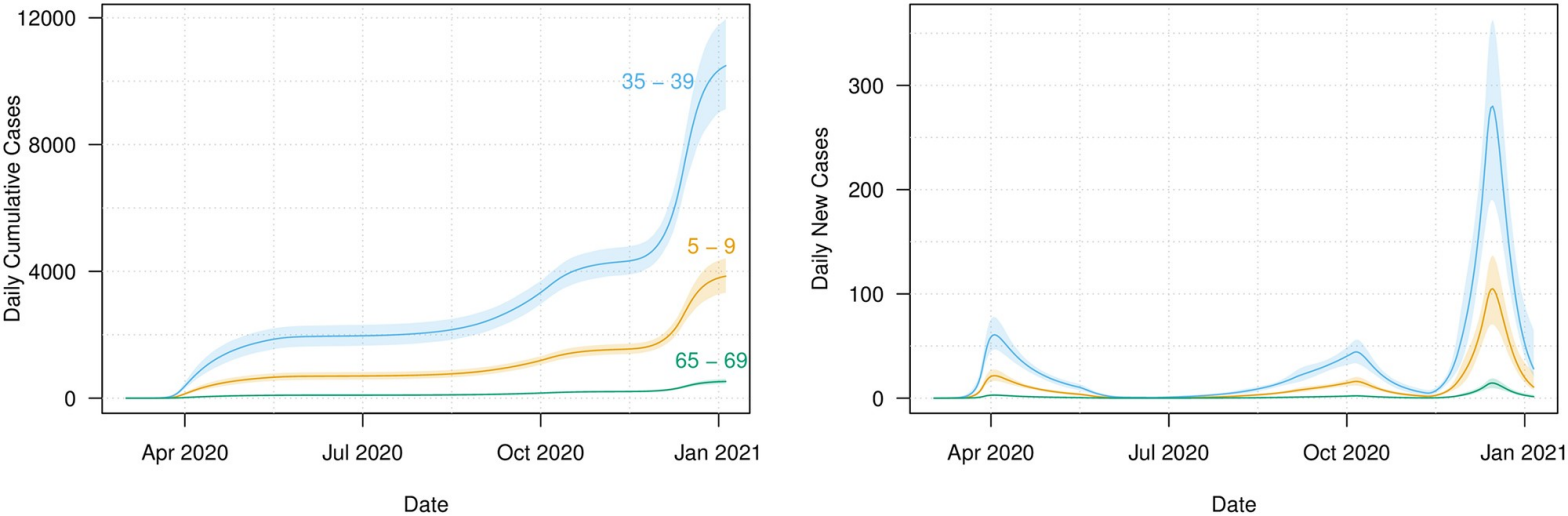
Case numbers for selected age classes obtained from the forward simulations of the SEIR with 95% intervals from bootstrapping. The age classes are 5–9 (orange), 35–39 (blue), and 65–69 (green).

Fig 4(a) shows the model fit to the daily cumulative cases data (i.e., $X(t_{d};\hat{\theta })$ and *x*_*d*_ respectively), while Fig 4(b) shows a plot of the model fit to the daily new cases data (i.e., $X\left(t_{d};\hat{\theta }\right)-X\left(t_{d-1};\hat{\theta }\right)$ and *x*_*d*_ − *x*_*d*−1_ respectively). Both figures illustrate that the model provides a good fit to the observed data albeit with slight deviations in the early days of the epidemic and later around the December holiday and New Year period. We observe from the model fit to daily new cases that these periods were characterised by larger variability in the daily recorded number of new cases. The presence of outliers may be as a result of data reporting issues, especially over the December holiday period. For example, in Ireland a testing backlog developed over this period with test results from multiple days being subsequently batched together. An advantage of our age-structured model is the availability of a breakdown of cases by age-group and in fact all compartments. The mixing patterns for each age group determine its rate of infection which in turn determines the number of observed cases for each age group. Fig 5 demonstrates the differences in model-based case numbers across selected age groups (see also S2 and S3 Figs in S1 File). We can see that the case numbers are higher for the middle-aged group (35–39) than for the younger (5–9) or older (65–69) groups, and note from Fig 2 that the number of contacts is higher for the middle-aged group.

**Fig 6:**
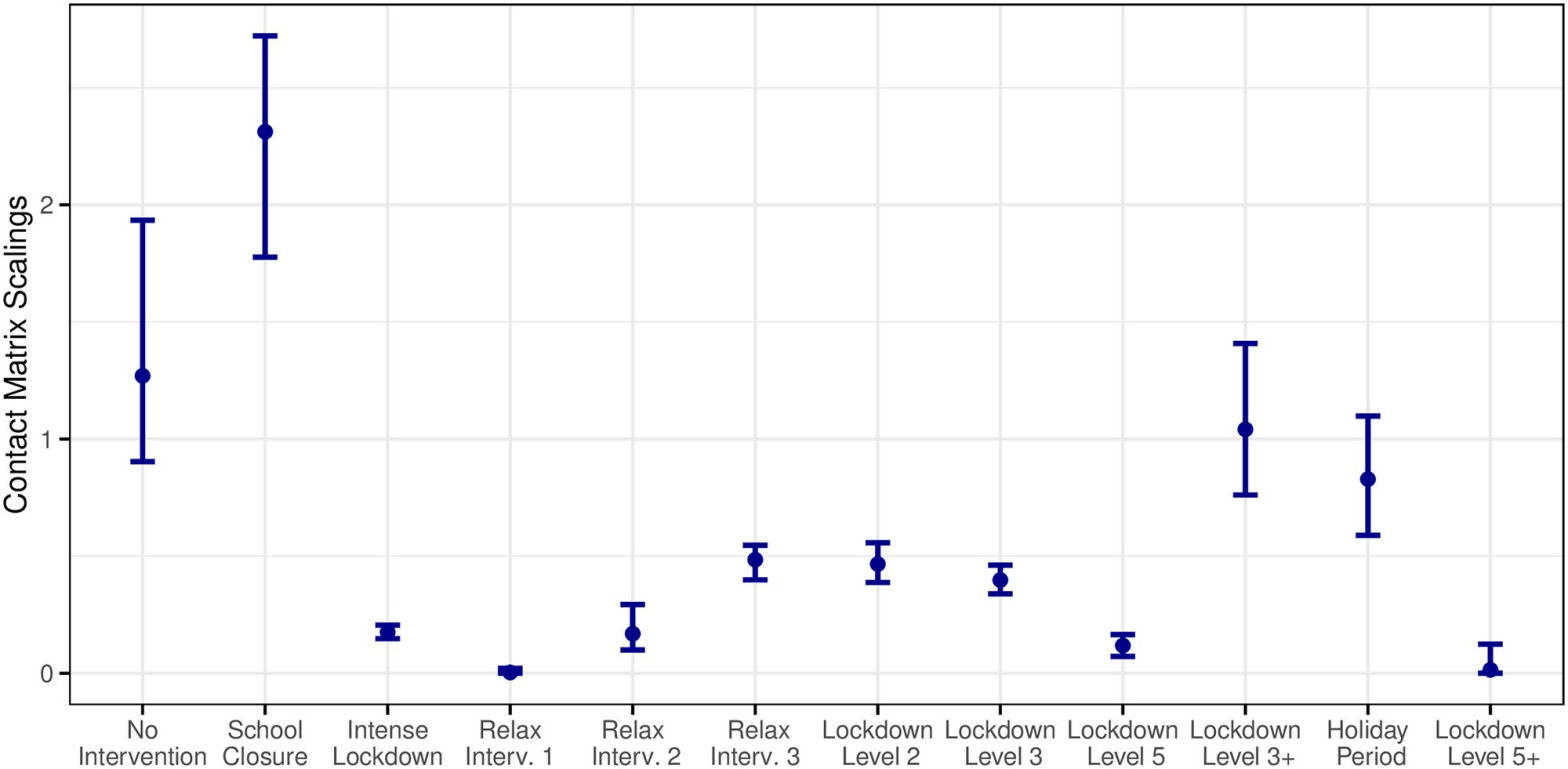
Estimates of the contact matrix scaling parameters for each lockdown period with 95% uncertainty intervals obtained through bootstrapping.

Fig 6 displays the estimated scaling parameters ***θ*** with bootstrapped 2.5% and 97.5% percentiles for each government policy observed over the period of study. The associated numeric values are given in S5 Table in S1 File.

The scaling parameter estimate for the no-intervention period, ${\hat{\theta }}_{1}$ ≈ 1.27 (95% confidence interval (CI) 0.90–1.94), indicates that the social contact patterns based on the POLYMOD study may be slightly under-estimating the current Irish contact patterns. Perhaps somewhat surprisingly, the parameter ${\hat{\theta }}_{2}$, corresponding to an initial school closure period, is greater than one (95% CI 1.78–2.72). However, this might be explained by the fact that it corresponds to a short period of time where no other measures had yet been introduced (apart from pub closures later in the period), but with an imminent government announcement of a strict nationwide lockdown expected—in line with what had been observed in other countries already by this stage in the global pandemic. During this period there was frenzied panic buying and stock piling of goods, increased travel across the country, and possibly increased social gatherings prior to movement restrictions. The remaining scaling parameters behave as expected based on the level of restrictions in place at that time: the higher the levels of restrictions, the smaller the scaling parameter, corresponding to reduced social mixing. The confidence intervals just prior to and including the holiday period indicate that social mixing returned to a near normal level at that time where a dramatic spike in the case numbers was also observed. This was followed immediately by a heavy lockdown and consequent drop in case numbers; indeed, the scaling parameter for this final period has the smallest value of all.

Over the period of study, note that we have witnessed two lockdown Level 3 periods (September/October and December 2020). However, although in theory both periods were designated as “Lockdown Level 3” by the Irish government, we have applied two separate scaling parameters for these two periods as the December lockdown included some relaxations compared to a full Level 3 lockdown. Specifically, non-essential retail and services were open once again and indoor service in restaurants and cafes was also permitted. This was done to facilitate people’s social needs around the holiday period, and indeed we see that the estimated parameter value for lockdown Level 3+ (December) is much larger than that for lockdown Level 3 (September/October). Again because of modifications in the execution, we have separate scaling parameters for the lockdown Level 5 in October/November and what we call the lockdown Level 5+ January 2021; the latter was stricter following the large rise in case numbers during the holiday period, and this is reflected in the small scaling value for this period as previously mentioned. Since the lockdown levels commenced with (what we label as) a Level 2 in August 2020, we have not observed a Level 1 or 4 lockdown. Prior to August 2020, the lockdowns and relaxations were more ad-hoc and do not fit into any particular governmental lockdown level. The contact matrices corresponding to the policy interventions based on our fitted model are displayed in S4 Fig in S1 File.

**Fig 7:**
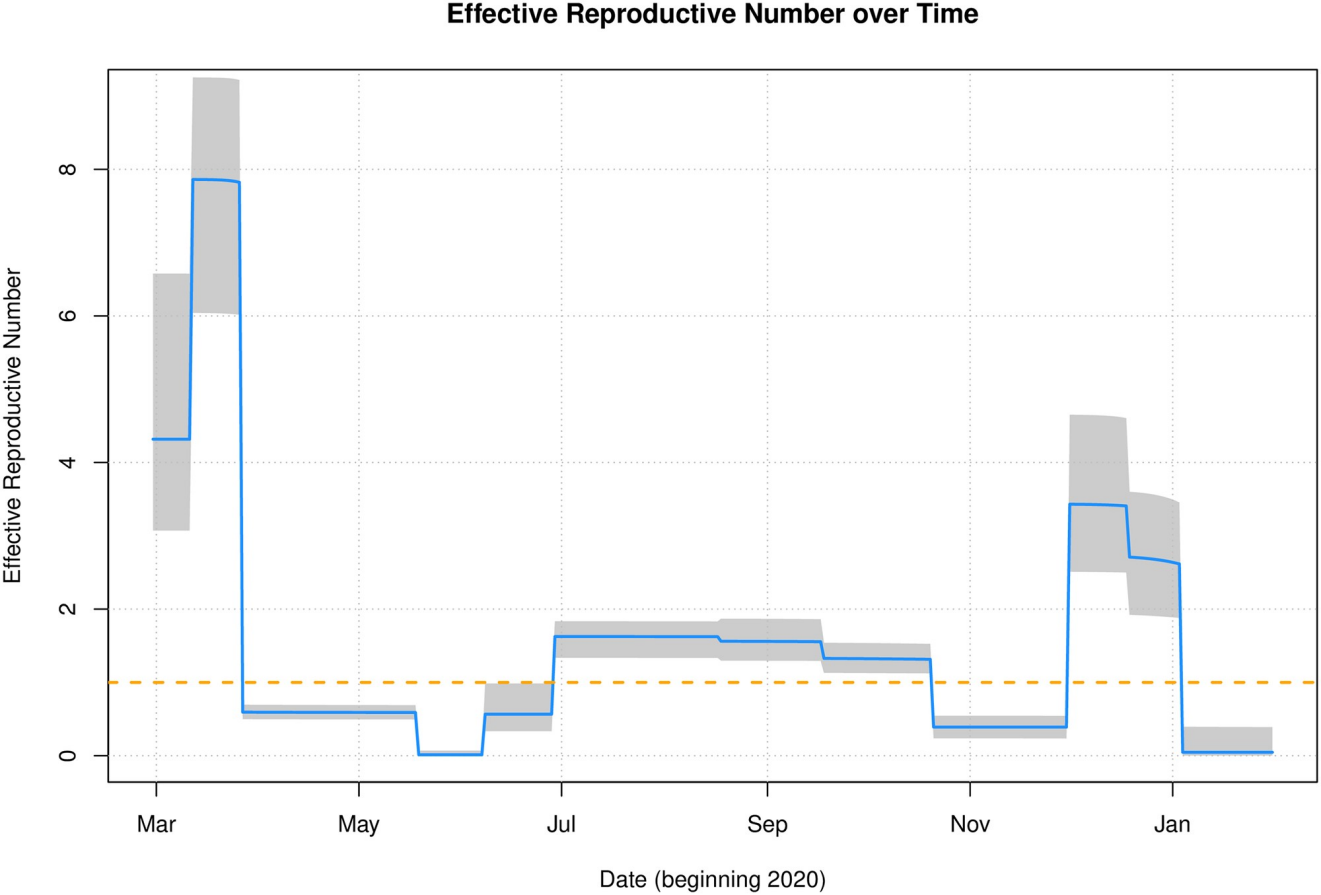
The effective reproduction number *R*(*t*). The solid line was calculated from the best fit parameters, and the uncertainty intervals were drawn by computing *R*(*t*) for each bootstrap replicate and selecting the 2.5% and 97.5% quantiles at each time point.

An important epidemiological metric is the *effective reproductive number*, *R*(*t*), the expected number of secondary infections at time *t*. It differs from *R*_0_, the *baseline* reproduction number, in that it changes over time and takes into account that the whole population will not be fully susceptible. A common estimate of *R*(*t*) is the product of *R*_0_ and the total proportion susceptible in the population at time *t* (see for example Section 2.2 of [41]). In the context of our model, the baseline reproductive number is *R*_0_ *θ*_*k*_ (rather than just *R*_0_) to account for the rate at which individuals interact with each other; recall from Section 2.3 that *θ*_*k*_ is the scaling parameter corresponding to the time interval $I_{k}=\left(r_{k},r_{k+1}\right]$. So our estimate of the effective reproductive number is

$$\begin{array}{c} R\left(t\right)=R_{0}\theta_{k}\tilde{S}\left(t\right) \end{array}$$ (6)

where $\tilde{S}\left(t\right)=\sum_{i=1}^{A}S_{i}\left(t\right)/\sum_{i=1}^{A}N_{i}\left(t\right)$ is the proportion of susceptible individuals in the the entire population at time *t*. Fig 7 displays the estimate of *R*(*t*) based on our fitted model. We see here that prior to the first heavy lockdown in April, *R*(*t*) was initially very large. This dropped below one following that first lockdown, but gradually increased again over the summer period when relaxations were introduced. It was brought back under control with successive Level 3 and Level 5 lockdowns, but markedly increased over the run-up to the December holiday period; *R*(*t*) was then driven towards zero with the lockdown Level 5+.

### 5.2 Assessing the economic cost of policy interventions

The approach of [26] provides us with a trained model to estimate weekly GDP growth during the pandemic. The inputs to the model are the Δ*s*_*l*,*w*_, year on year differences in weekly log search intensities on Google Trends for *l* = 1, …, 24 search terms over *w* = 1, …, 52 weeks. The model has a country specific fixed effect resulting in country targeted forecasts (denoted here by “ire” for Ireland). The inputs are passed to a nonlinear function $\hat{f}\left(\cdot ,\cdot \right)$, which has been trained using historic data as described in Section 2.4. This results in

$$\begin{array}{c} {\hat{g}}_{\text{ire},w}=\hat{f}({\left\{\Delta s_{l,w}\right\}}_{l,w},“\text{ire}”) \end{array}$$

where ${\hat{g}}_{\text{ire},w}$, is a forecast of GDP growth for week *w* in Ireland.

**Fig 8:**
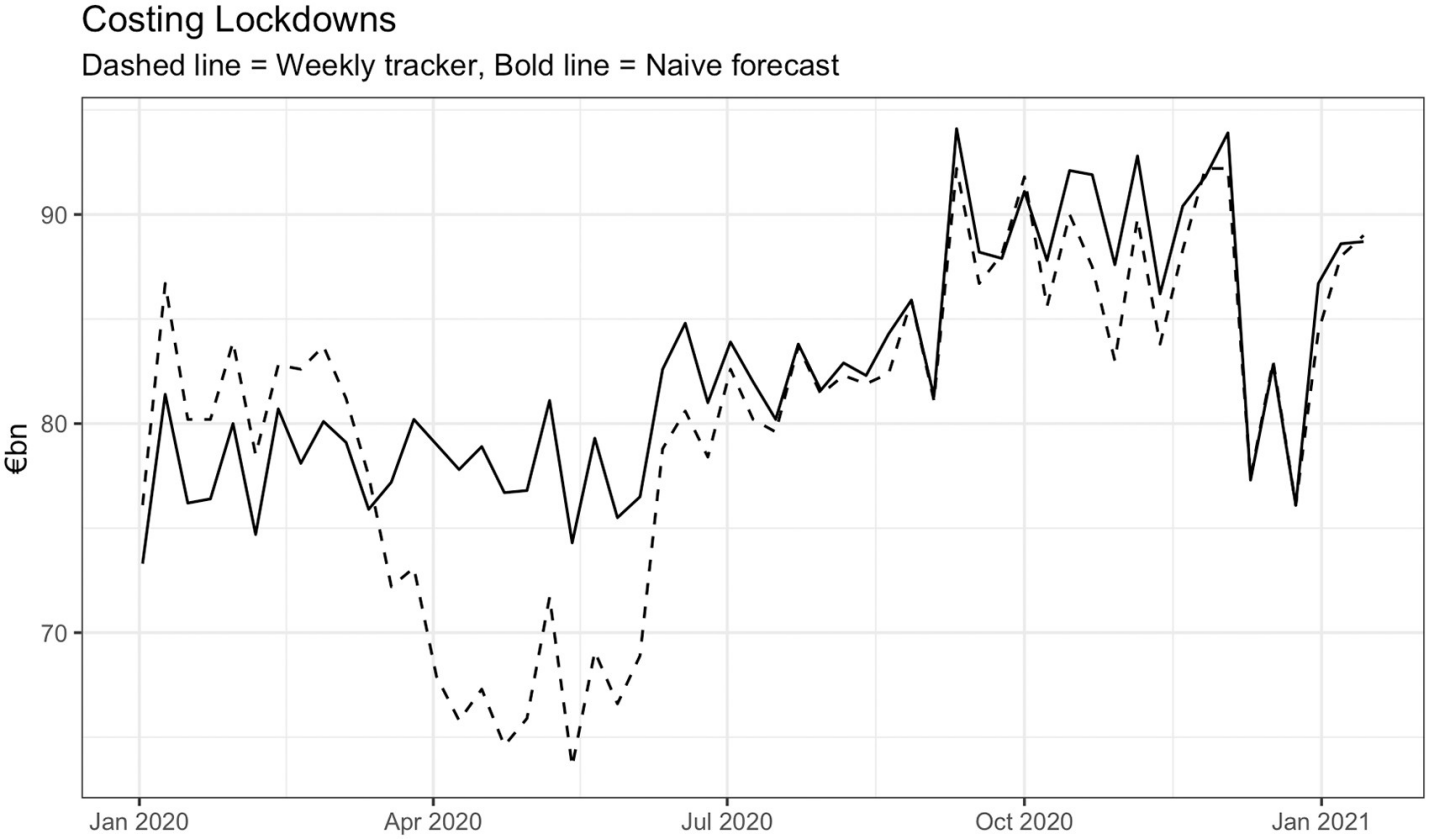
Comparison of weekly GDP estimates in Ireland for the naive no-pandemic ARIMA(1, 1, 1) (bold) and weekly tracker (dashed) approaches.

Assuming weights equal to 1/48 (due to a 48 week working year) and an average counterfactual annualised growth of 5.8% given by a naive forecast, the impact on growth rates per week can be calculated as $\frac{1}{48}\left(5.8-{\hat{g}}_{\text{ire},w}\right)$. Multiplying these by 2019 measures of GDP in euros gives an estimated cost per week in nominal euros. A counter-factual “no COVID” scenario can be constructed by naively forecasting the 2019 estimated weekly growth rate forward using an ARIMA(1, 1, 1) model to provide ${\tilde{g}}_{\text{ire},w}$, with GDP in nominal euros is estimated in the same way. Fig 8 shows the weekly estimates of GDP for each approach, with the difference in weekly GDP figures between the approaches suggesting an estimate of the economic cost per week of pandemic related measures. Linking estimated weekly costs to the lockdown strategies implemented results in the estimated economic costs shown in Table 2.

**Table 2:** Estimated economic costs for each lockdown period.

Start	End	Policy	Cost (€bn)
29/02/2020	11/03/2020	No Intervention	-2.47
12/03/2020	26/03/2020	School Closure	-3.68
27/03/2020	18/05/2020	Intense Lockdown	37.14
19/05/2020	07/06/2020	Relax Intervention 1	-11.74
08/06/2020	28/06/2020	Relax Intervention 2	5.07
29/06/2020	17/08/2020	Relax Intervention 3	4.77
18/08/2020	17/09/2020	Lockdown Level 2	3.69
18/09/2020	20/10/2020	Lockdown Level 3	0.90
21/10/2020	30/11/2020	Lockdown Level 5	8.03
01/12/2020	18/12/2020	Lockdown Level 3+	1.30
19/12/2020	03/01/2021	Holiday period	1.67
04/01/2021	31/01/2021	Lockdown Level 5+	5.00

This simple analysis shows that relative to the counterfactual “no COVID” experiment, the majority of cost to society was incurred during the first intense lockdown period. Recoveries tended to overshoot the naive forecasted values (hence the negative readings in Table 2 for the relaxation of interventions) for a number of reasons. For example, pent up demand in the household sector, once released, is very easily produced and consumed. The economy was also supported by large-scale government intervention which began at scale in May and June of 2020 involving government injection of cash directly into the economy to support households and firms. Participants in the economy adapted and adjusted to their changed circumstances. This is why the calculation of elasticities in [26] is important to include. Once people moved their consumption online, and government supports were in place, the impact of the crisis was substantially attenuated.

## 6 Forward projection and counterfactual scenario analysis

A key strength of our modelling framework is that it enables forward projection, and quantification of projection uncertainty, of anticipated case numbers under specific health interventions. In the following we simulate epidemiological and economic costs of a number of counterfactual scenarios based on hypothetical policy decisions taken by the Irish Government. We compare these to observed epidemiological data for that period to evaluate predictive performance. S6 Table in S1 File presents the Irish Health Protection and Surveillance Centre’s (HPSC’s) total number of cases and deaths up to 28^th^ December 2020 [42]. The data was no longer broken down by age category to the same granularity after that date. From this we can establish approximate estimates of death rates for each age class that we can apply to the forecasts of the SEIR model.

### 6.1 Projected cases, economic costs and deaths

Evaluation of the health impact of government restrictions on population mobility requires a contact scaling matrix for lockdown levels 1 to 5. However, as described in Section 5.1, Level 1 and Level 4 lockdowns have not been used to date (and hence not observed), while Level 3 and Level 5 lockdowns were used twice but with modifications on each of the second occasions. Moreover, seasonal shifts in social dynamics (for example, in December), may play a role in the effectiveness of a given lockdown. Our chosen scaling estimates for projection, based on S4 Table in S1 File, make a pragmatic best approximation. For Level 0 (No Intervention), we use the ‘No intervention’ scaling from the fitted model; Level 1 (unobserved) uses a linear interpolation of scalings from Level 0 and 2; Level 3 was enacted twice with slight changes the second time, but we use the scaling from the October 2020 lockdown (‘Lockdown Level 3’); Level 4 was unobserved, so we use again a linear interpolation, this time from Level 3 to Level 5; Level 5 was used twice, but similar to Level 3 case, we use the scaling from November 2020 corresponding to ‘Lockdown Level 5’. The resulting scaling parameter estimates mapped to lockdown levels are presented in S6 Fig in S1 File for completeness, and for reference we list values in S5 Table in S1 File.

**Fig 9:**
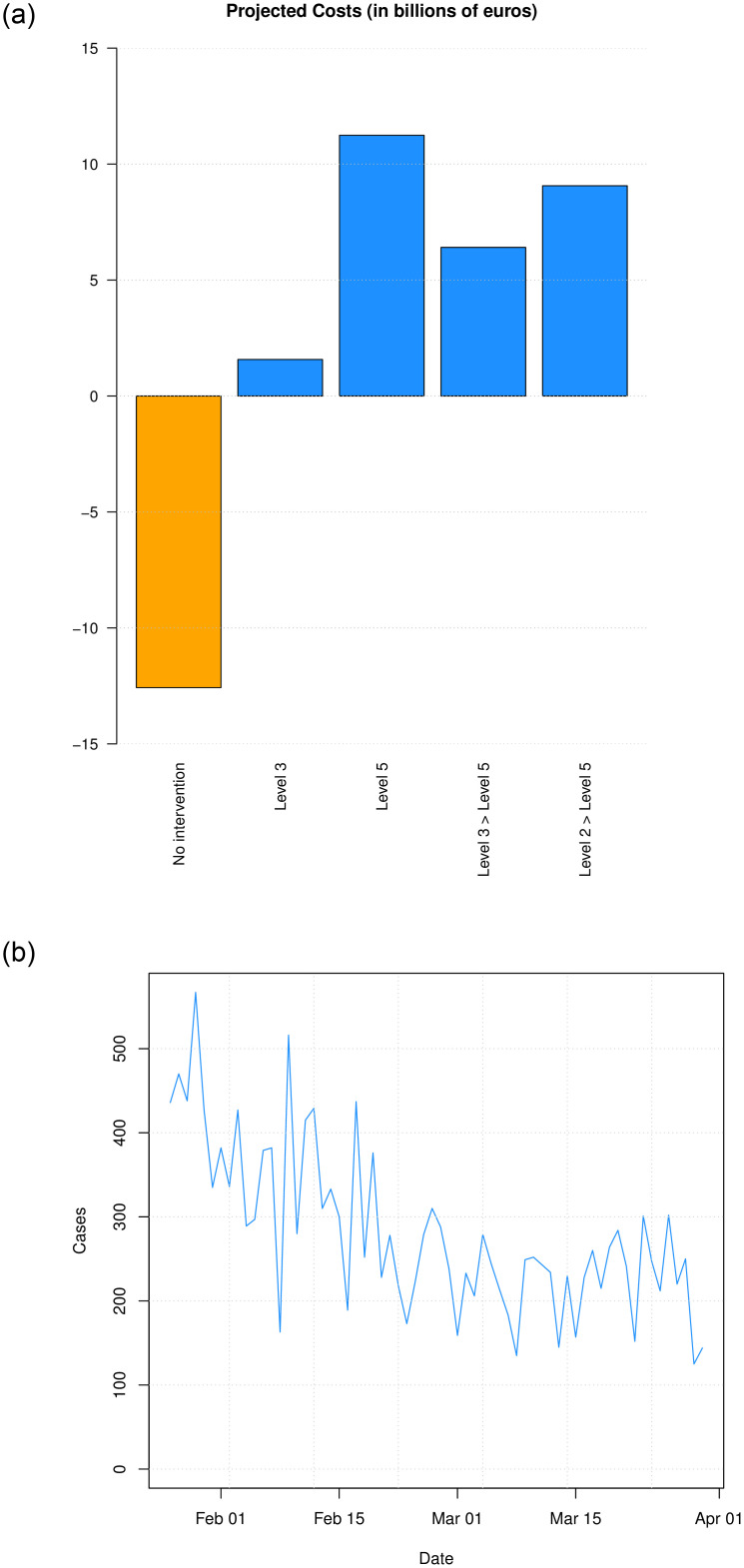
Left: Estimated economic costs for the 8 week period between 1st February 2021 and 30th March 2021. Right: Time series of actual daily cases for 8 week period between 1st February 2021 and 30th March 2021.

The SEIR model was projected from the 1st of February to 30th March 2021 (8 weeks) under different lockdown scenarios implemented in two 4 week chunks (the date of the second lockdown implemented was 2nd March). Lockdown levels used for each four week period are specified in Table 3. A forward projection is made by adopting the corresponding scaling of contacts from S5 Table in S1 File, with the SEIR system solved forward in time. Projection uncertainty bounds are obtained by taking the bootstrap replicate estimates of the corresponding scaling parameters and completing a forward projection for each of these, with 2.5% and 97.5% sample quantiles of these projections giving uncertainty bounds. Estimated daily economic costs are shown in the left panel of Fig 9.

**Table 3:** Estimated cases, deaths and costs for the 8 week period between 1st February 2021 and 30th March 2021. Where there are two scenarios listed, the shift occurred on the 2nd March. The observed number of cases in the given period was 15,334 for the Dublin region. 95% intervals are given in parentheses.

Lockdown Scenario	Estimated cases / 100 (Lower, Upper)	Estimated deaths (Lower, Upper)	Estimated cost (€bn)
No Intervention	5073 (1797, 8735)	2785 (881, 7230)	-12.6
Level 3	122 (78, 307)	51 (34, 121)	1.6
Level 5	25 (16, 72)	17 (12, 39)	11.2
Level 3 → Level 5	87 (60, 211)	41 (29, 92)	6.4
Level 2 → Level 5	118 (75, 306)	53 (35, 129)	9.1

An estimate of the number of deaths was obtained by taking the product of the proportions from S6 Table in S1 File and the corresponding age group from the daily removed count, estimated by

$$\begin{array}{c} \frac{I^{\text{SI}}+I^{\text{SN}}}{\tau_{\text{D}}-\tau_{\text{C}}+\tau_{\text{L}}}+\frac{I_{i}^{\text{PI}}}{\tau_{\text{D}}-\tau_{\text{C}}+\tau_{\text{L}}-\tau_{\text{R}}} \end{array}$$ (7)

for each day and age group. The cost, case and death estimates are shown in Table 3. While the daily COVID-19 case incidence information for the Dublin region is available, the daily death data is not provided at this level of geographic granularity.

**Fig 10:**
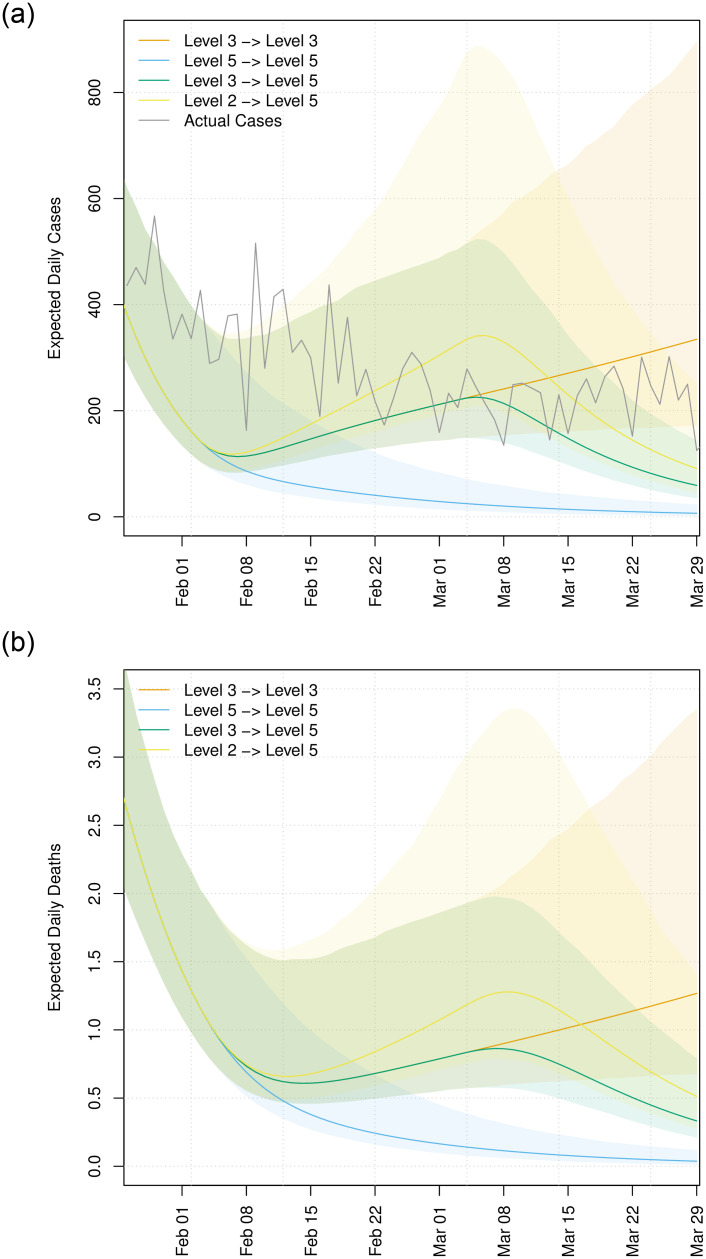
Projected infection rates and deaths under different lockdown scenarios. The death data is not available for the Dublin region for this period.

During this time, Ireland was under Level 5 lockdown. This corresponds to our third lockdown scenario above, though as can be seen in Fig 10, the projection underestimates the true case count of 15, 334 that was observed during that period. In contrast, the upper bound of our estimated number of daily cases during this period is approximately 7, 200. This underestimation may be due to the public not treating this Level 5 as seriously as the Level 5 intervention last year. A closer look at the actual case counts over that period is given in the right panel of Fig 9, showing that cases plateaued after February 15th oscillating around an average of approximately 200 cases per day instead of falling. A comparison across the different interventions of the total number of estimated cases for the period between 1st February 2021 and 30th March 2021 is shown in S7 Fig in S1 File.

## 7 Discussion

In this article we propose a modelling framework that attempts to answer the question of how best public health strategies can be weighed against the attendant social and economic costs of such measures, taking into account age-specific contact rates. We have presented an age-structured SEIR model for the Irish epidemic that can be deployed to an international context with the appropriate data. Where feasible, the parameters of our model governing disease spread have been estimated from publicly available Irish epidemiological data with a bootstrapping approach used to determine parameter uncertainties. Our fitted model captures much of the structure observed in daily case numbers. Our approach allows for local adaptions and calibrations of models in any region or location where such data is available. We also present incidence projections under a number of hypothetical government intervention strategies, in conjunction with approximate economic costings allowing for their consideration in the decision making process. This framework is easy to interpret and suitable for describing counterfactual scenarios, which could assist policy makers with regard to minimising morbidity balanced with the costs of prospective suppression strategies. We are not aware of any framework with this level of modelling detail that has been presented to date, especially in the Irish context.

However, there are a number of limitations to our present approach that provide opportunities for substantial further refinements. These chiefly revolve around access to sufficiently detailed public health and economic data which enable the development of more complex models around social mixing, in addition to further refinement of estimation of parameters characterising disease spread and economic costings.

We currently assume that the disease spread parameters are uniform across all age groups. This is a simplifying assumption but a necessary one due to the unavailability of such information for the Irish population and the limited literature on such parameters elsewhere at the time of writing this article. Whenever this information becomes available, our proposed model can be extended straightforwardly to incorporate age-dependent disease spread parameters. We have also assumed that the effect of the non-pharmaceutical interventions is uniform across age-groups. This could be relaxed by allowing age-dependent scaling parameters for all interventions as follows

$$\begin{array}{c} (\begin{array}{cccc} \theta_{k1} & 0 & \ldots & 0\\ 0 & \theta_{k2} & \ldots & 0\\ \vdots & \vdots & \ddots & 0\\ 0 & 0 & \ldots & \theta_{kA} \end{array})(\begin{array}{ccc} c_{1\;1} & \ldots & c_{1\;A}\\ c_{2\;1} & \ldots & c_{2\;A}\\ \vdots & \ldots & \vdots \\ c_{A\;1} & \ldots & c_{A\;A} \end{array})=(\begin{array}{ccc} \theta_{k1}c_{1\;1} & \ldots & \theta_{k1}c_{1\;A}\\ \theta_{k2}c_{1\;1} & \ldots & \theta_{k2}c_{2\;A}\\ \vdots & \ldots & \vdots \\ \theta_{kA}c_{A\;1} & \ldots & \theta_{kA}c_{A\;A} \end{array}),\;k=1,\ldots ,N. \end{array}$$

Here, the non-zero elements of the leading diagonal matrix represent the effect of lockdown on each age group, and these are free parameters which would need to be inferred. Thus, with *A* = 16 (age groups) and *N* = 12 (lockdown periods), this extension yields almost 200 parameters to be estimated. We can simplify slightly by adding constraints on some diagonal elements to be equivalent (for example, group them into young/middle/old) or perhaps use a regularisation approach to reduce the effective number of estimated parameters, but even this may be over-reaching for shorter lockdown periods where there is very little data. Such approaches might be more feasible if the daily case data were available broken down by each age, as it could essentially be viewed as 16 separate optimisation problems. However, notwithstanding the fact that such data are not publicly available in Ireland, at such a fine scale we would expect to have sparse case count data for some age groups wherein estimation of the intervention effects would be challenging.

We build upon the social interaction matrices provided by [21] which are confined to four social settings, where we model changes due to public mobility restrictions through a rescaling approach. However, with contact tracing for confirmed cases being used as a control strategy in a number of countries, access to such data would provide an avenue to substantially improve social mixing models, perhaps in conjunction with carefully constructed large scale public mobility surveys. For example, we could expand the number of social mixing venues to more than four with a better understanding on disease transmission settings. This would allow for incorporation of specific venues such as bars or restaurants. Alternatively, we could model the sociability parameters using covariates to describe the specific lockdown (e.g., schools open/closed, pubs open/closed, public events, restrictions in households etc.) rather than fixing these to have a constant value in a given lockdown level. This would allow us to make comparisons between Level 5 and Level 5+ for example—a holiday period effect could be included as another covariate. It would also allow us to construct new hypothetical lockdown regimes.

Another extension to our approach would be to incorporate uncertainty estimates around the mechanistic parameters of the SEIR model. If one could obtain reasonable uncertainty intervals on these parameters, one direction might be to use a central composite design scheme used in response surface construction [43], based on some transformation of these uncertainty intervals. However, such an approach would introduce a steep computational overhead and would require sufficient coding and hardware solutions to enable a feasible (in time) implementation. If attention is focused on a small subset of mechanistic parameters then the problem is less demanding and this can be handled instead by straightforward bootstrapping, for example [12] fits a model and quantifies uncertainty via bootstrapping when only allowing *R* to vary. Another option is to include these parameters in the optimisation alongside the contact matrix scales, while imposing heavy box constraints on the range of candidate values for these parameters within the optimisation, as used by [35]. Another potential complication with modelling infectious diseases is their propensity to mutate over time. More infectious strains will manifest in the data as rising case numbers that we can feed into the model but if it evolves rather drastically, as seems to be the case with the Delta variant [44], then it may be necessary to re-evaluate the parameters.

We have extended the model developed by IEMAG, the Irish national modelling group whose work has informed government throughout the pandemic. Our proposed approach moves closer to reality by incorporating age-specific social dynamics, and, consequently, provides a more flexible framework in which age-specific infection characteristics could also be included whenever these are better understood. Further extensions, as discussed in the preceding paragraphs, are also possible within this framework but are beyond the scope of the current article; these will be a focus of future work.

## Supporting information

S1 File The supporting information file includes S1–S7 Figs and S1–S6 Tables, in addition to some additional explanation and discussion. (ZIP)
