# Supplementary material for: An age-structured SEIR model for COVID-19 incidence in Dublin, Ireland with framework for evaluating health intervention cost
Source: PLoS One. 2021 Dec 7;16(12):e0260632. doi: 10.1371/journal.pone.0260632 (PMC8651129; doi:10.1371/journal.pone.0260632)
Supplement: S1 File — (ZIP) [file pone.0260632.s001.zip › Covid19_SupplementaryInfo.pdf]

# **Supplementary material:** An age-structured SEIR model for COVID–19 incidence in Dublin, Ireland with framework for evaluating health intervention cost

## Appendix A: Detailed table of lockdown measures in Ireland

A more detailed overview of the government interventions over the course of the epidemic in Ireland from what we define as day 0 of the epidemic, 28th February 2020, up to and including 31st January 2021. We acknowledge that certain interventions were not eased for the whole indicated period but for the purpose of simplification we are assuming they were.

| Start | End | Government Intervention |
|---|---|---|
| 29/02/2020 | 11/03/2020 | No Intervention |
| 12/03/2020 | 26/03/2020 | All schools and universities closed, followed by bars and a ban on mass gathering (although there was a four day delay between the schools closure and the closure of the bars, we decided to treat it as the same period for simplification) |
| 27/03/2020 | 18/05/2020 | A strict lockdown whereby all non-essential services and industries were put on hold |
| 19/05/2020 | 07/06/2020 | The first phase of the gradual easing of the strict lockdown |
| 08/06/2020 | 28/06/2020 | The second phase of the gradual easing of the strict lockdown |
| 29/06/2020 | 17/08/2020 | The third phase of the gradual easing of the strict lockdown |
| 18/08/2020 | 17/09/2020 | Some level of restrictions was brought back, although the government's plan of living with COVID–19 and the introduction of the 5 levels lockdown tiers was introduced later during this phase, the restrictions were very similar to those of what the government now call a Level 2 lockdown, hence we label the intervention implemented during this phase as a Level 2 lockdown. |
| 18/09/2020 | 20/10/2020 | Level 3 lockdown |
| 21/10/2020 | 30/11/2020 | Level 5 lockdown |
| 01/12/2020 | 18/12/2020 | Level 3 lockdown, all non-essential retail, hairdressers, gyms, leisure centres, museums, galleries, libraries, cinemas and places of worship were allowed to reopen. Restaurants, gastropubs and hotel restaurants were allowed to provide indoor service. |
| 19/12/2020 | 03/01/2021 | Holiday period, households were allowed to mix with up to two other households and inter-county travel was permitted. |
| 04/01/2021 | 30/01/2021 | Level 5 lockdown, plus the closure of schools and the construction industry. |

**Table S1.** A summary of the government interventions that were implemented over the study time frame.

## Appendix B: Next generation matrix

The next generation matrix encodes the spread of the disease as a linear operator whose form is determined by the model. [1] showed that (subject to light conditions) the dominant eigenvalue of the *next-generation operator* can be interpreted as "the typical number of secondary cases", or $R_0$. For *discrete* state models such as ours, Section 2.2 of [2] provides a practical explanation of how to construct the next generation *matrix.* We go through the method here for our model.

Let $\tilde{\mathbf{z}} = (\tilde{z}_1, \ldots, \tilde{z}_p)$ be the vector of compartment sizes for compartments from which infected individuals enter or leave (i.e, all except the susceptible and removed compartments). The entries of $\tilde{\mathbf{z}}$ are extracted from $\mathbf{z}$ as defined in Section 3 of the manuscript. Now introduce $f_i(\tilde{\mathbf{z}})$ as the rate of *new* infections that enter compartment $i$, let $v_i^+(\tilde{\mathbf{z}})$ be the rate of individuals arriving into compartment $i$ who are *not* newly infected, let $v_i^-(\tilde{\mathbf{z}})$ be the rate of individuals leaving compartment $i$, and finally let $v_i(\tilde{\mathbf{z}}) = v_i^-(\tilde{\mathbf{z}}) - v_i^+(\tilde{\mathbf{z}})$. With this notation, every system of equations described in section 3 of the main manuscript can be expressed as $f_i(\tilde{\mathbf{z}}) - v_i(\tilde{\mathbf{z}})$.

The next generation matrix is constructed from the matrices of partial derivatives of $f_i$ and $v_i$,

$$F_{ij} = \frac{\partial f_i}{\partial \tilde{z}_j}(\tilde{\mathbf{z}}_0), \qquad V_{ij} = \frac{\partial v_i}{\partial \tilde{z}_j}(\tilde{\mathbf{z}}_0)$$

evaluated at the disease-free equilibrium, $\tilde{\mathbf{z}}_0$, i.e., the point at which no infection is present. In our application, since we are imposing a constant population $N$, the disease free equilibrium simply means that $S_i = N_i, i = 1, \ldots, A$ and all other compartments equal 0. The next generation matrix $\mathbf{Q}$ is equal to

$$\mathbf{Q} = \mathbf{F}\,\mathbf{V}^{-1}$$

In our application, the $\mathbf{F}$ matrix can be expressed as a block matrix, where each block corresponds to an age group

$$\mathbf{F} = \begin{bmatrix} \mathbf{F}_{11} & \mathbf{F}_{12} & . & . & . & \mathbf{F}_{1A} \\ \mathbf{F}_{21} & \mathbf{F}_{22} & . & . & . & \mathbf{F}_{2A} \\ . & . & . & & & . \\ . & . & & . & & . \\ . & . & & & . & . \\ \mathbf{F}_{A1} & \mathbf{F}_{A2} & . & . & . & \mathbf{F}_{AA} \end{bmatrix}$$

where each block is given by

$$\mathbf{F}_{mn} = \begin{bmatrix} 0 & \alpha B_{mn} & B_{mn} & \kappa B_{mn} & B_{mn} & \kappa B_{mn} & B_{mn} \\ 0 & 0 & 0 & 0 & 0 & 0 & 0 \\ 0 & 0 & 0 & 0 & 0 & 0 & 0 \\ 0 & 0 & 0 & 0 & 0 & 0 & 0 \\ 0 & 0 & 0 & 0 & 0 & 0 & 0 \\ 0 & 0 & 0 & 0 & 0 & 0 & 0 \\ 0 & 0 & 0 & 0 & 0 & 0 & 0 \end{bmatrix}.$$

Note that only the first row of each block is non-zero and $B_{mn}$ is defined as follows

$$B_{mn} = \beta c_{mn} \frac{N_m}{N_n}.$$

Beware that the subscripts $m$ and $n$ here correspond to the block indices, not its cell position in the matrix. Assuming there is no movement between the age groups the $\mathbf{V}$ matrix is expressed as a block diagonal matrix where each block corresponds to an age group.

$$
\mathbf{V} = \begin{bmatrix} \mathbf{V}_{11} & 0 & . & . & . & 0 \\ 0 & \mathbf{V}_{22} & . & . & . & 0 \\ . & . & . & & & . \\ . & . & & . & & . \\ . & . & & & . & . \\ 0 & 0 & . & . & . & \mathbf{V}_{AA} \end{bmatrix}
$$

Each block is given by

$$
\mathbf{V}_{ii} = \begin{bmatrix} \frac{1}{\tau_{\mathrm{L}}} & 0 & 0 & 0 & 0 & 0 & 0 \\ -\frac{p_{\mathrm{AS}}}{\tau_{\mathrm{L}}} & \frac{1}{\tau_{\mathrm{D}}} & 0 & 0 & 0 & 0 & 0 \\ -\frac{1-p_{\mathrm{AS}}}{\tau_{\mathrm{L}}} & 0 & \frac{1}{\tau_{\mathrm{C}}-\tau_{\mathrm{L}}} & 0 & 0 & 0 & 0 \\ 0 & 0 & -\frac{p_{\mathrm{SI}}}{\tau_{\mathrm{C}}-\tau_{\mathrm{L}}} & \frac{1}{\tau_{\mathrm{D}}-\tau_{\mathrm{C}}-\tau_{\mathrm{L}}} & 0 & 0 & 0 \\ 0 & 0 & -\frac{p_{\mathrm{T}}}{\tau_{\mathrm{C}}-\tau_{\mathrm{L}}} & 0 & \frac{1}{\tau_{\mathrm{R}}} & 0 & 0 \\ 0 & 0 & -\frac{1-p_{\mathrm{SI}}-p_{\mathrm{T}}}{\tau_{\mathrm{C}}-\tau_{\mathrm{L}}} & 0 & 0 & \frac{1}{\tau_{\mathrm{C}}-\tau_{\mathrm{D}}+\tau_{\mathrm{L}}} & 0 \\ 0 & 0 & 0 & 0 & -\frac{1}{\tau_{\mathrm{R}}} & 0 & \frac{1}{\tau_{\mathrm{D}}-\tau_{\mathrm{C}}+\tau_{\mathrm{L}}-\tau_{\mathrm{R}}} \end{bmatrix}
$$

Since each block represents an age group, different parameters for different age can be easily incorporated by simply altering the suitable block.

The largest absolute eigenvalue value of $\mathbf{Q}$ is $R_0$. Since $\beta$ is easily factored out of the $\mathbf{F}$ matrix, the eigenvalue can be expressed as a product of $\beta$ and the maximum eigenvalue of $\widehat{\mathbf{F}}\,\mathbf{V}^{-1}$, where $\mathbf{F} = \beta\,\widehat{\mathbf{F}}$. This means that if $R_0$ is determined and $\beta$ is desired, the expression can easily be re-arranged:

$$
\beta = R_0/\xi
$$

where $\xi$ is the maximum eigenvalue of $\widehat{\mathbf{F}}\,\mathbf{V}^{-1}$.

## Appendix C: Exploring the impact of age-structuring and mixing

To examine the impact of population mixing matrices on the modelling, a number of hypothetical contact matrix scenarios are investigated. First, due to the relatively homogeneous contact matrix specified for Ireland by [3] (S1 Figure), the results obtained from our age structured approach do not differ substantially from the model without age structuring, and this is not surprising as our specification for the impact of government interventions scales linearly. In contrast, we show the impact on parameter inference through three synthetic contact matrices. Matrix 1 scales the [3] contact matrix by 1/2; matrix 2 is constructed through a randomly selecting either 0 or 3 for each entry of the matrix; matrix 3 groups the age classes into three groups, allowing for contact between the age classes within each group but not between (as per the [3] contact matrix). The synthetic matrices and the contact matrix based on [3] can be seen in S1 Figure with the resulting parameter estimates in S2 Table. Note that the parameter estimates and fit (sum of squares) do vary with respect to the contact matrices.

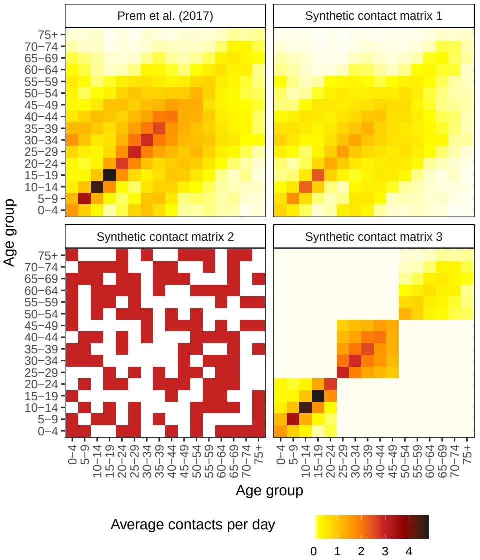


**Fig S1.** The contact matrix from the [3] study and the synthetic contact matrices we create.

| | [3] matrix | Synthetic matrix 1 | Synthetic matrix 2 | Synthetic matrix 3 |
|---|---|---|---|---|
| No Intervention | 1.270 | 1.414 | 0.912 | 2.948 |
| School Closure | 2.312 | 5.757 | 0.895 | 3.182 |
| Intense Lockdown | 0.176 | 0.376 | 0.063 | 0.253 |
| Relax Intervention 1 | 0.003 | 0.277 | 0.185 | 0.201 |
| Relax Intervention 2 | 0.169 | 0.012 | 0.124 | 0.192 |
| Relax Intervention 3 | 0.485 | 0.649 | 0.115 | 0.662 |
| Lockdown Level 2 | 0.466 | 1.773 | 0.329 | 0.686 |
| Lockdown Level 3 | 0.398 | 0.553 | 0.186 | 0.610 |
| Lockdown Level 5 | 0.118 | 0.561 | 0.052 | 0.212 |
| Lockdown Level 3+ | 1.041 | 0.749 | 0.601 | 1.427 |
| Holiday period | 0.829 | 2.332 | 0.351 | 1.342 |
| Lockdown Level 5+ | 0.015 | 0.005 | 0.022 | 0.007 |
| Total Sum of Squares | 79405587 | 184946468 | 160243281 | 81893893 |

**Table S2.** Estimated contact scaling parameters and $RSS$ value for models with different assumed contact matrices (matrix 1,2,3) S1 Figure.

## Appendix D: Additional Tables and Figures

| Parameter | Description | Value | Reference |
|---|---|---|---|
| $\tau_{\mathrm{C}}$ | Average incubation period | 5.8 | [4] |
| $\tau_{\mathrm{P}}$ | Average pre-symptomatic period | 2 | [5] |
| $\tau_{\mathrm{L}}$ | Average latent period. This is computed as $\tau_{\mathrm{C}}$ minus $\tau_{\mathrm{P}}$ | 3.8 | |
| $\tau_{\mathrm{D}}^{C}$ | Average infectious period for symptomatic patients | 13.4 | [5] |
| $\tau_{\mathrm{D}}^{SC}$ | Average infectious period for asymptomatic patients | 6 | [5] |
| $\tau_{\mathrm{D}}$ | Average infectious period. Weighted average of the symptomatic and asymptomatic periods (weighted by prevalence) | 13.5 | |
| $R_0$ | Basic reproductive number | 3.4 | [6] |
| $\alpha$ | Factor reduction of transmission from asymptomatic cases | 0.55 | [7] (The mean of given interval) |
| $\kappa$ | Factor reduction of transmission from isolating cases | 0.05 | [8] |
| $p_{\mathrm{AS}}$ | Proportion of asymptomatic infections | 0.20 | [9] |
| $p_{\mathrm{T}}$ | Proportion symptomatic who get tested | 0.8 | [8] |
| $p_{\mathrm{SI}}$ | Proportion symptomatic who self-isolate | 0.1 | [8] |
| $\tau_{\mathrm{R}}$ | Expected time between first symptoms and test result | 7 | [8] |

**Table S3.** List of parameters used directly in or sourced to for the specification of the SEIR model.

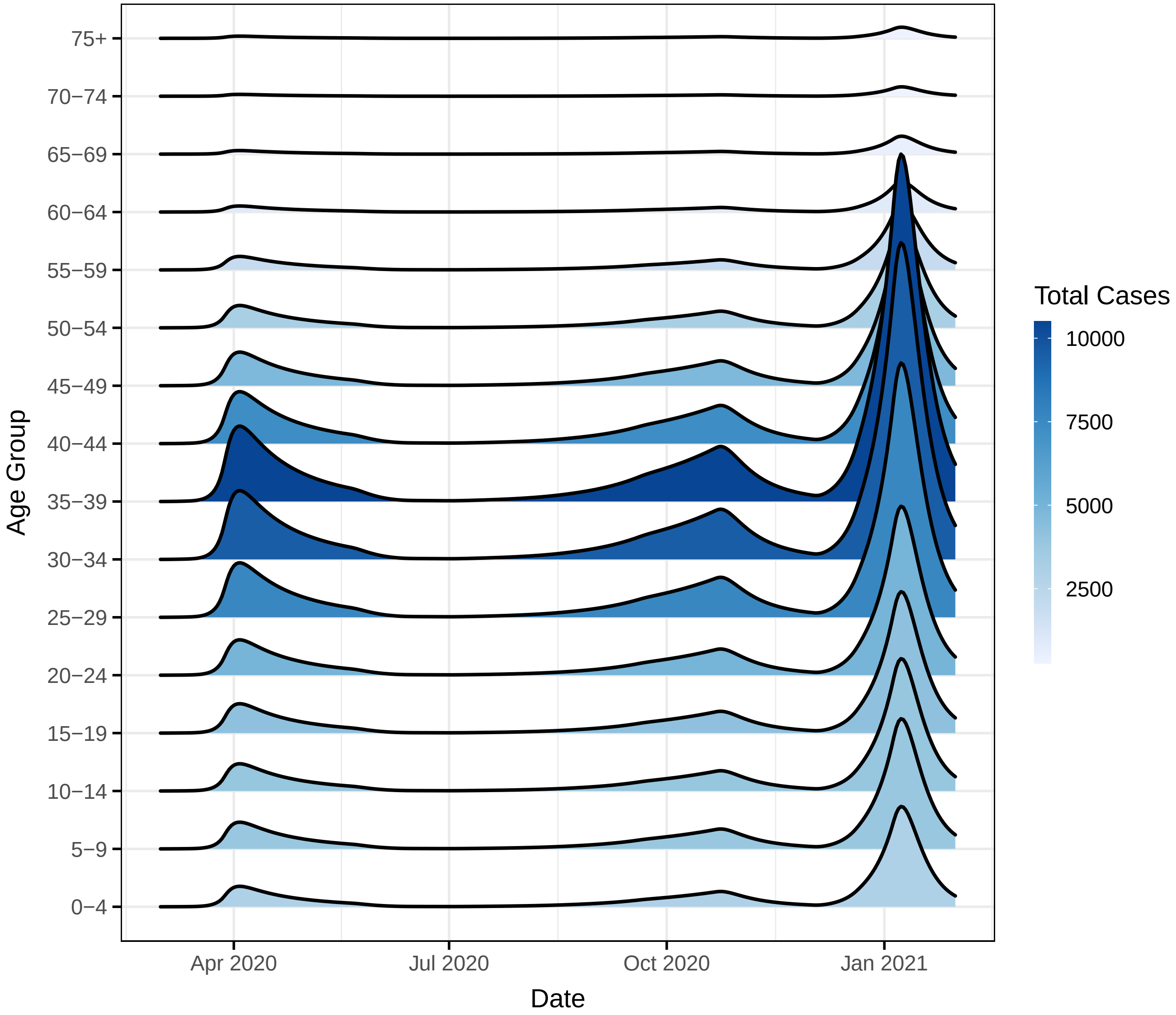


**Fig S2.** The model fit daily case counts for each age group over the study period.

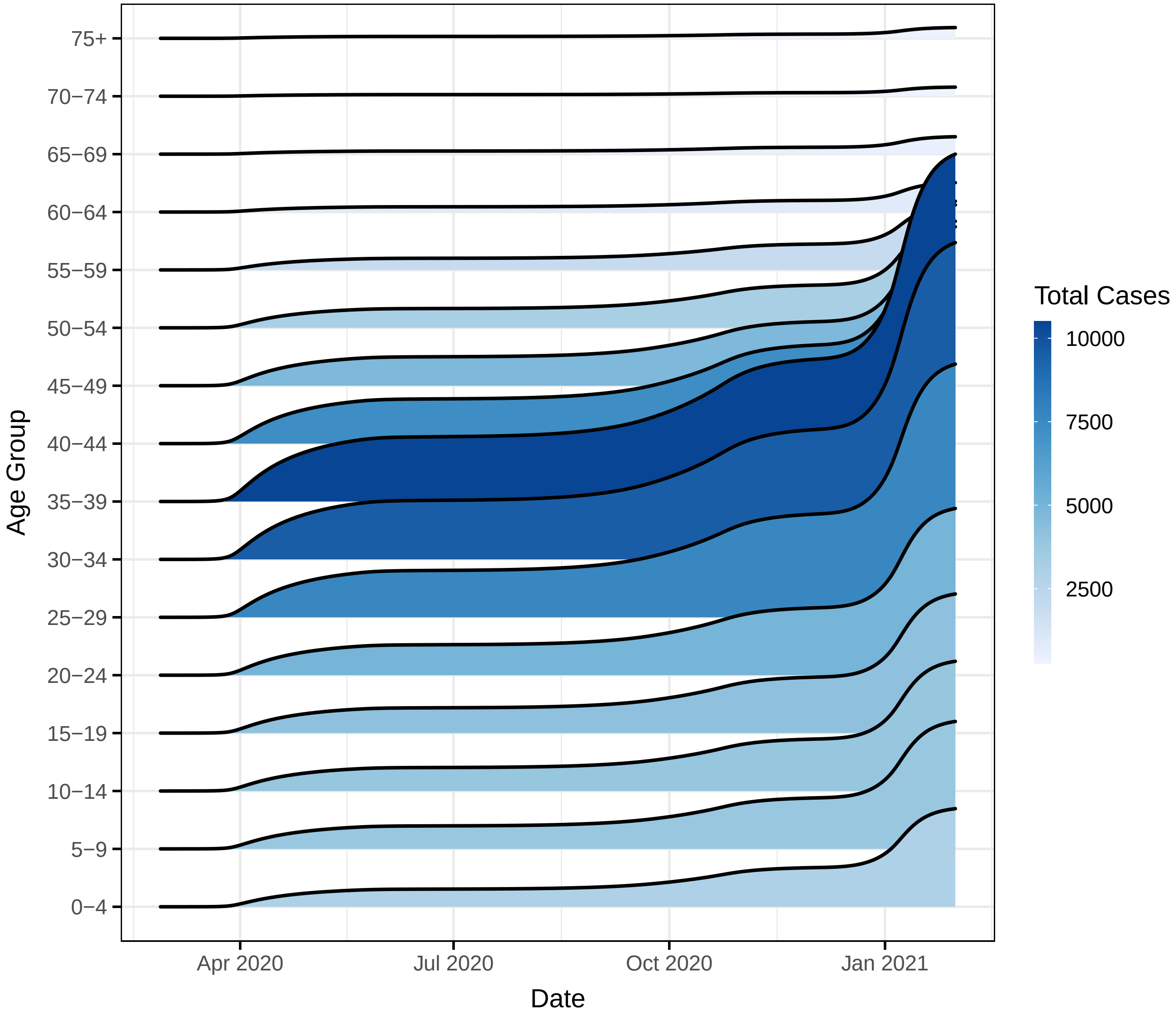


**Fig S3.** The model fit cumulative case counts for each age group over the study period.

| Policy | $\widehat{\boldsymbol{\theta}}$ | Median | Lower Bound | Upper Bound |
|---|---|---|---|---|
| No Intervention | 1.270 | 1.342 | 0.904 | 1.935 |
| School Closure | 2.312 | 2.244 | 1.777 | 2.722 |
| Intense Lockdown | 0.176 | 0.175 | 0.148 | 0.205 |
| Relax Intervention 1 | 0.003 | 0.004 | $< 0.001$ | 0.021 |
| Relax Intervention 2 | 0.169 | 0.187 | 0.100 | 0.293 |
| Relax Intervention 3 | 0.485 | 0.470 | 0.399 | 0.546 |
| Lockdown Level 2 | 0.466 | 0.471 | 0.388 | 0.557 |
| Lockdown Level 3 | 0.398 | 0.398 | 0.339 | 0.462 |
| Lockdown Level 5 | 0.118 | 0.117 | 0.072 | 0.165 |
| Lockdown Level 3+ | 1.041 | 1.046 | 0.761 | 1.408 |
| Holiday period | 0.829 | 0.812 | 0.589 | 1.099 |
| Lockdown Level 5+ | 0.015 | 0.016 | $< 0.001$ | 0.124 |

**Table S4.** The estimated contact matrix scaling parameters for each **observed** lockdown phase, with bootstrapped 95% uncertainty bounds.

| Policy | Scalars | Median | Lower Bound | Upper Bound |
|---|---|---|---|---|
| Lockdown Level 0 | 1.270 | 1.342 | 0.904 | 1.935 |
| Lockdown Level 1 | 0.868 | 0.908 | 0.691 | 1.200 |
| Lockdown Level 2 | 0.466 | 0.471 | 0.388 | 0.557 |
| Lockdown Level 3 | 0.398 | 0.398 | 0.339 | 0.462 |
| Lockdown Level 4 | 0.258 | 0.258 | 0.232 | 0.286 |
| Lockdown Level 5 | 0.118 | 0.117 | 0.072 | 0.165 |

**Table S5.** The estimated scaling parameters for each of the lockdown levels specified by the Irish Government.

| Age Range | Total Cases | Total Deaths | Proportion of Deaths to Cases |
|---|---|---|---|
| 0-14 | 8552 | 0 | 0 |
| 15-24 | 15505 | $< 5$ | $< 3.2 \times 10^{-4}$ |
| 25-34 | 15120 | 6 | $3.9 \times 10^{-4}$ |
| 35-44 | 13900 | 14 | $10^{-3}$ |
| 45-54 | 13176 | 38 | $2.9 \times 10^{-3}$ |
| 55-64 | 9445 | 94 | 0.01 |
| 65-74 | 4977 | 332 | 0.07 |
| 75+ | 7734 | 1736 | 0.22 |

**Table S6.** HPSC's COVID–19 total case and death rate in Ireland up to 28th December 2020 (this was the latest point that the HPSC reported the full cumulative death rates with these age groups). Where there were more than 0 but less than 5 cases in a cell, the HPSC did not report the number to retain anonymity. There were a further 30 cases where the age was not known.

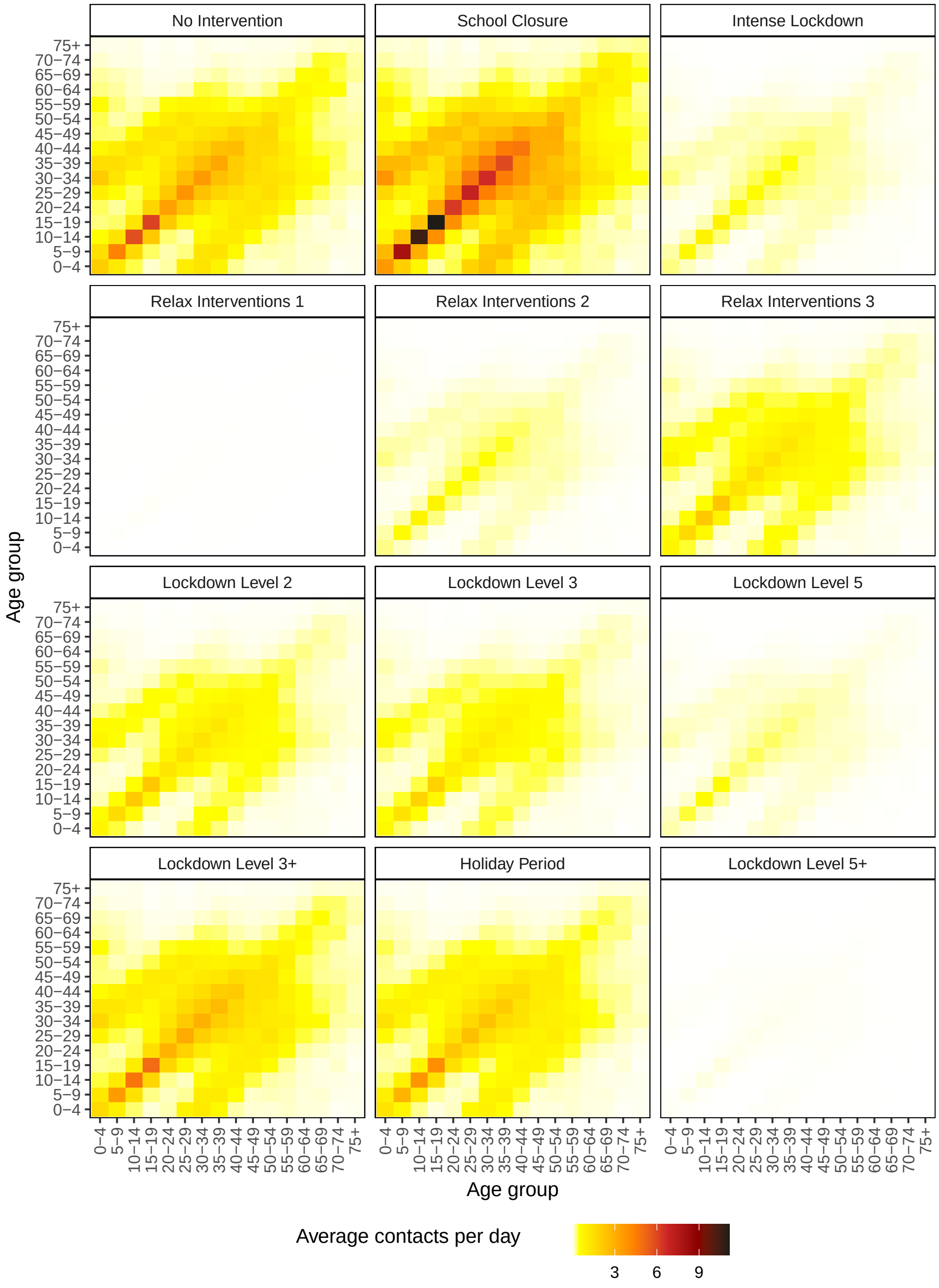


**Fig S4.** The estimated contact matrix for each of the policies witnessed over the study time frame.

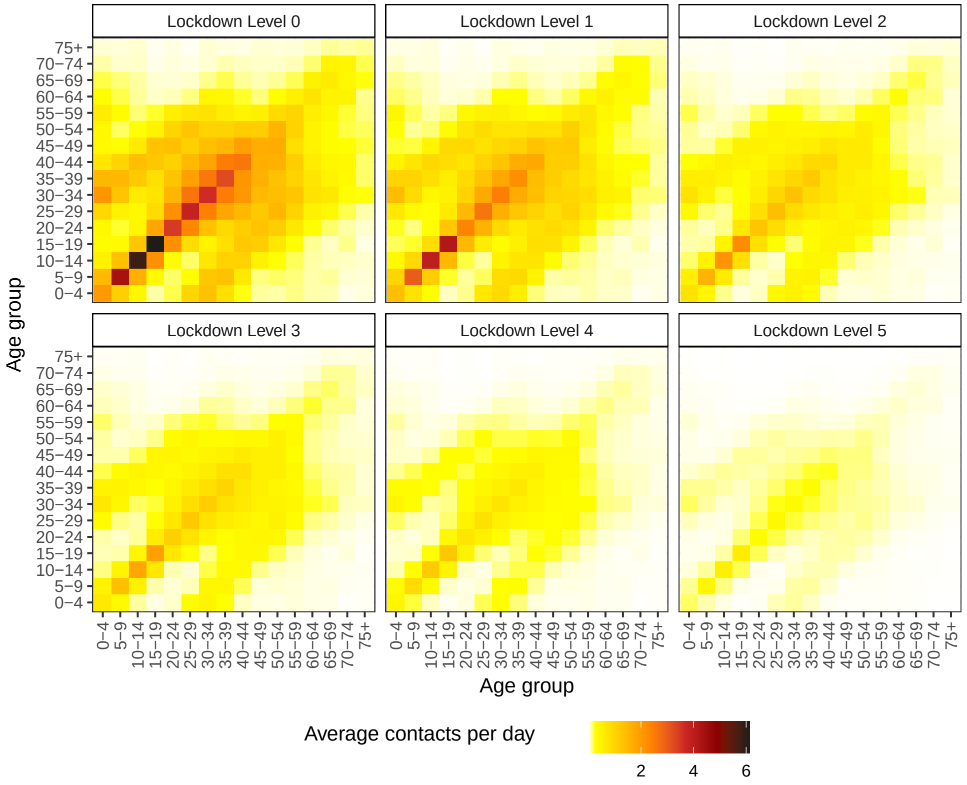


**Fig S5.** Estimated contacts matrix for each of the 5 lockdown levels specified by the Irish Government.

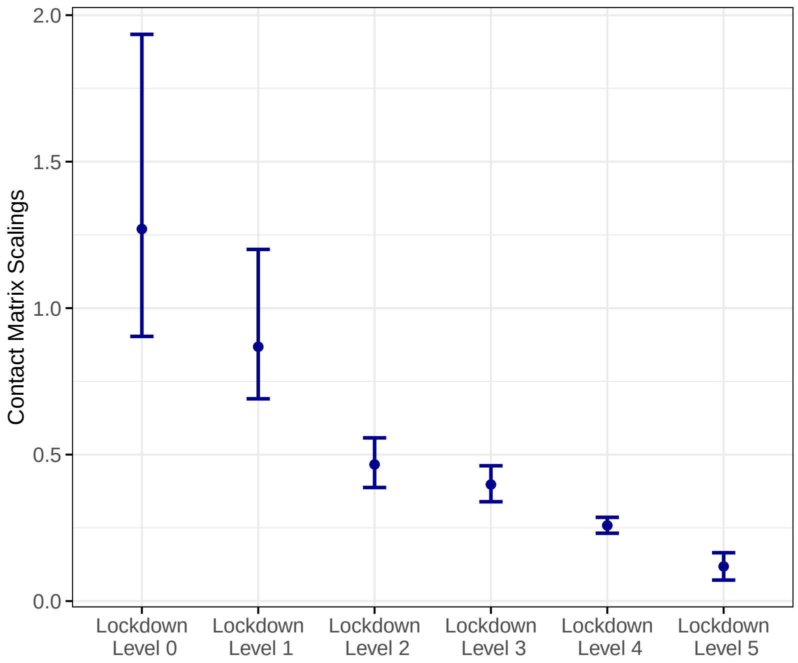


**Fig S6.** The estimated contact matrix scaling parameters for Government specified projections.

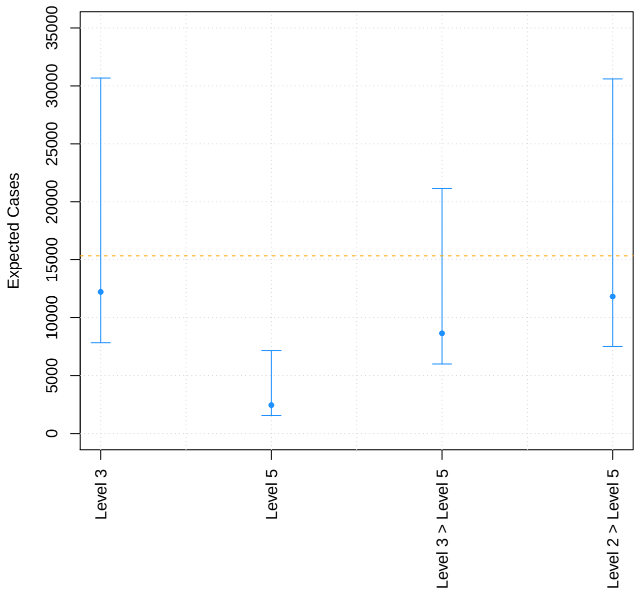


**Fig S7.** Estimated total cases for the 8 week period between 1st February 2021 and 30th March 2021. 'No Intervention' has been omitted as it as the scale of its estimated cases is substantially larger than the others.

# References


1. Diekmann O, Heesterbeek JAP, Metz JAJ. On the Definition and the Computation of the Basic Reproduction Ratio R0 in Models for Infectious Diseases in Heterogeneous Populations. Journal of Mathematical Biology. 1990;28(4):365–382. doi:10.1007/BF00178324.

2. Heffernan JM, Smith RJ, Wahl LM. Perspectives on the Basic Reproductive Ratio. Journal of the Royal Society Interface. 2005;2(4):281–293. doi:10.1098/rsif.2005.0042.

3. Prem K, Cook AR, Jit M. Projecting Social Contact Matrices in 152 Countries Using Contact Surveys and Demographic Data. PLOS Computational Biology. 2017;13(9):e1005697. doi:10.1371/journal.pcbi.1005697.

4. McAloon C, Collins Á, Hunt K, Barber A, Byrne AW, Butler F, et al. Incubation Period of COVID-19: A Rapid Systematic Review and Meta-Analysis of Observational Research. BMJ Open. 2020;10(8):e039652. doi:10.1136/bmjopen-2020-039652.

5. Byrne AW, McEvoy D, Collins AB, Hunt K, Casey M, Barber A, et al. Inferred Duration of Infectious Period of SARS-CoV-2: Rapid Scoping Review and Analysis of Available Evidence for Asymptomatic and Symptomatic COVID-19 Cases. BMJ Open. 2020;10(8):e039856. doi:10.1136/bmjopen-2020-039856.

6. Náraigh LÓ, Byrne Á. Piecewise-Constant Optimal Control Strategies for Controlling the Outbreak of COVID-19 in the Irish Population. Mathematical Biosciences. 2020;330:108496. doi:10.1016/j.mbs.2020.108496.

7. Evoy DM, McAloon CG, Collins AB, Hunt K, Butler F, Byrne AW, et al. The Relative Infectiousness of Asymptomatic SARS-CoV-2 Infected Persons Compared with Symptomatic Individuals: A Rapid Scoping Review. medRxiv. 2020; p. 2020.07.30.20165084. doi:10.1101/2020.07.30.20165084.

8. IEMAG. A Population-Level SEIR Model for COVID-19 Scenarios. Irish Department of Health; 2020. Available from: `www.hse.ie`.

9. Buitrago-Garcia D, Egli-Gany D, Counotte MJ, Hossmann S, Imeri H, Ipekci AM, et al. Occurrence and Transmission Potential of Asymptomatic and Presymptomatic SARS-CoV-2 Infections: A Living Systematic Review and Meta-Analysis. PLOS Medicine. 2020;17(9):e1003346. doi:10.1371/journal.pmed.1003346.
